# Removal of Cr(VI) from aqueous solution using ball mill modified biochar: multivariate modeling, optimization and experimental study

**DOI:** 10.1038/s41598-024-55520-9

**Published:** 2024-02-28

**Authors:** Yunfeng Tan, Jinxia Wang, Lingling Zhan, Hongjun Yang, Yinchun Gong

**Affiliations:** 1 College of River and Ocean Engineering, Chongqing Jiaotong University, Chongqing, 400074 China; 2 College of Resources and Safety, Chongqing Vocational Institute of Engineering, Chongqing, 402260 China; 3 General College, Chongqing Vocational Institute of Engineering, Chongqing, 402260 China; 4 College of Resources and Environment, Southwest University, Chongqing, 400715 China; 5 Chongqing Zhihai Technology Co., Ltd, Chongqing, 402260 China

**Keywords:** Environmental chemistry, Environmental impact

## Abstract

Chromium (Cr(VI)) pollution has attracted wide attention due to its high toxicity and carcinogenicity. Modified biochar has been widely used in the removal of Cr(VI) in water as an efficient and green adsorbent. However, the existing biochar prepared by chemical modification is usually complicated in process, high in cost, and has secondary pollution, which limits its application. It is urgent to explore modified biochar with simple process, low cost and environmental friendliness. Therefore, ball milling wheat straw biochar (BM-WB) was prepared by ball milling technology in this paper. The adsorption characteristics and mechanism of Cr(VI) removal by BM-WB were analyzed by functional group characterization, adsorption model and response surface method. The results showed that ball milling effectively reduced the particle size of biochar, increased the specific surface area, and more importantly, enhanced the content of oxygen-containing functional groups on the surface of biochar. After ball milling, the adsorption capacity of Cr(VI) increased by 3.5–9.1 times, and the adsorption capacity reached 52.21 mg/g. The adsorption behavior of Cr(VI) follows the pseudo-second-order kinetics and Langmuir isotherm adsorption model rate. Moreover, the Cr(VI) adsorption process of BM-WB is endothermic and spontaneous. Under the optimized conditions of pH 2, temperature 45 °C, and adsorbent dosage 0.1 g, the removal rate of Cr(VI) in the solution can reach 100%. The mechanism of Cr(VI) adsorption by BM-WB is mainly based on electrostatic attraction, redox and complexation. Therefore, ball milled biochar is a cheap, simple and efficient Cr(VI) removal material, which has a good application prospect in the field of remediation of Cr(VI) pollution in water.

## Introduction

Chromium (Cr) is a typical non-biodegradable heavy metal^1^. In nature, chromium mainly exists in the form of Cr(III) and Cr(VI)^2^. It is widely used in various industrial processes, such as electroplating, leather tanning, steelmaking and mining^3,4^. Among them, Cr(VI) is highly mobile in soil and aquatic systems, and its toxicity, mutagenicity and carcinogenicity are 500 times that of Cr(III)^5,6^, and its mutagenicity is about 1000 times that of Cr(III). It has been listed by the United States Environmental Protection Agency as one of the 17 chemicals that pose a major threat to humans^7^. In recent years, due to the frequent occurrence of agricultural product safety accidents caused by Cr(VI) pollution, the natural environment and residents’ health are threatened, which has become one of the main environmental problems. Therefore, it is very important to find an efficient, environmentally friendly, economical, simple and feasible method to repair chromium pollution in water and soil.

In 2022, the total output of wheat in the world was as high as 780 million tons, of which the annual output of wheat in China was as high as 138 million tons, accounting for 18% of the world’s total output. Its production occupies the core position of the world. The ratio of waste to grain is usually 1.3:1, so the production of wheat straw waste is as high as 180 million tons per year^8^. As an attractive renewable biological resource, although wheat straw is used as feed for animal husbandry and fuel for various industrial boilers, more part of it is used for burning in the field, dumping in forest areas and natural biodegradation or not used at all. In order to make better use of these waste straws, this study is based on wheat straw as raw material, which is converted into wheat straw biochar (WB) by pyrolysis. Its performance is similar to biochar, but the cost is cheaper. Many researchers have done various studies on the pyrolysis of wheat straw or other forms of biochar, and there is a big gap in the adsorption capacity of heavy metals in wastewater. Although China is the largest wheat producer, there are relatively few studies on the direct use of WB as an adsorbent for Cr(VI) adsorption in wastewater.

At present, the removal methods of Cr(VI) mainly include adsorption, chemical precipitation, ion exchange, electrochemical method and biotechnology^9,10^. Among them, adsorption method has the advantages of high efficiency, low cost and simple operation, and has been widely concerned in the remediation of heavy metal contaminated soil and water^11,12^. Among many adsorption materials, biochar has the characteristics of wide source of raw materials, low cost, large specific surface area, rich pore structure and many surface functional groups, and has certain adsorption capacity for heavy metal elements^13–16^. However, due to the poor pore structure and small specific surface area of the original biochar, the adsorption capacity of Cr(VI) is low^17^. Researchers often modify the biochar by physical or chemical treatment methods. By changing the surface functional groups, specific surface area and pore structure of the original biochar, the safety, efficiency, reusability and environmental friendliness of the biochar are improved, and the adsorption performance of the modified biochar is more efficient and stable^18–20^. Among these modification methods, ball milling is a new engineering technology and modification method to improve the physical and chemical properties of materials by mechanical force^21,22^. The modification of biochar by ball milling can increase the specific surface area of the original biochar, provide more active adsorption sites for Cr(VI), and act as an electronic shuttle to promote the reduction of Cr(VI) and accelerate the adsorption kinetics of Cr(VI)^23,24^. In addition, the ball milling method can also make the oxygen-containing functional groups be more conducive to the adsorption and reduction of Cr(VI) by changing the type and content of functional groups on the surface of biochar^24,25^. Carboxyl and hydroxyl groups can adsorb Cr(VI) through complexation, while alcohol-OH and phenol-OH can provide electrons in the redox process, directly reducing Cr(VI) to Cr(III), and oxidizing it to carbonyl (C=O) and quinone structure^26,27^. Although many researchers have done a lot of research in this area in recent years, the adsorption behavior of Cr(VI) on biochar surface functional groups is still unclear and needs further study. In order to fill this research gap, the Box–Behnken design (BBD) was used in the experimental design, which uses a set of mathematical and statistical techniques to model the process and helps to understand the interactions between the optimized parameters^28^. Compared with the traditional single-parameter optimization method, it can prevent excessive time, space and material consumption, thereby reducing the number of tests required^29,30^. In addition, the method evaluates the interaction effects of multiple factors in different ranges through three-dimensional graphics to determine the optimal conditions for Cr(VI) removal.

This study used ball-milling modified wheat biochar (BM-WB) as an adsorption material to explore the optimal operating parameters of BM-WB adsorption of Cr(VI) and the dynamic mechanism of the adsorption process. The effects of main parameters on the adsorption efficiency of BM-WB were studied by batch experiments. At the same time, the best fitting model was determined by isothermal, kinetic and thermodynamic studies, and the optimal conditions of five factors (pH, time, temperature, dosage and initial concentration) were determined by Box-Behnken design (BBD). The adsorption mechanism of Cr(VI) removal process was proposed by Brunauer–Emmet–Teller (BET) analysis, energy dispersive X-ray analysis (EDX) and element mapping analysis, Fourier transform infrared spectroscopy (FTIR), X-ray diffraction spectroscopy (XRD) and X-ray photoelectron spectroscopy (XPS).

## Materials and methods

### Experimental materials

The chemical drugs used in this study, such as potassium dichromate (K_2_Cr_2_O_7_, ≥ 99.8%), sulfuric acid (H_2_SO_4_, ≥ 95%), phosphoric acid (H_3_PO_4_, ≥ 85%), diphenyl carbazide (C_13_H_14_N_4_O) and hydrochloric acid (HCl, ≥ 36%) were provided by Chengdu Cologne Chemical Co., Ltd., and acetone (CH_3_COCH_3_, ≥ 99.5%) and sodium hydroxide (NaOH, ≥ 96%) were purchased from Chongqing Wansheng Chuandong Chemical Co., Ltd. All solutions were prepared with ultrapure water (Conductivity of 18.2 MΩ cm) (Labonova Direct Pro, Think-lab, Germany). Cr(VI) stock solution (1000 mg/L) was prepared by dissolving K_2_Cr_2_O_7_ in ultrapure water. The working solution of the required Cr(VI) concentration was prepared daily by appropriately diluting the reserve solution. Unless otherwise specified, the purity of the chemicals and reagents used in this experiment were analytically pure.

### Material preparation

Preparation of WB: The wheat straw used in this study was collected from Nanyang, Henan Province, and ground by a grinder. After passing through a 40-mesh sieve, it was placed in a vacuum tube furnace (OTF-1200X, Shenzhen Kejing Zhida Technology Co., Ltd., China); before the start of pyrolysis, high-purity nitrogen was introduced at a flow rate of 100 mL/min for 30 min to exhaust the residual air in the pyrolysis system. After the start of pyrolysis, the flow rate of nitrogen remains unchanged, the heating rate is 10 °C/min, and the furnace body is heated from room temperature to the target pyrolysis temperature of 300 °C for 2 h. After the pyrolysis, it was cooled to room temperature in a muffle furnace, and then the residue was recovered and the biochar was washed three times with ultrapure water and dried at 60 °C for 12 h. Sealed in a brown glass bottle for subsequent analysis. N_2_ (purity of 99.999%) was used in the pyrolysis process. The prepared sample was labeled as WB.

Preparation of BM-WB: 10 g WB and 100 g agate balls (8, 10, 15 mm in diameter) were mixed into agate bottles, and then the agate bottles were placed in a planetary ball mill (MITR-YXQM-2L, Changsha Miqi Instruments and Equipment Co., Ltd., China). Rotating at 1500 rpm for 60 min, passing 100-mesh sieve, sealed and stored in brown glass bottles for subsequent analysis. The prepared sample was labeled as BM-WB.

### Characterization of biochar

In this study, the specific surface area and pore properties of BM-WB materials were determined by nitrogen adsorption–desorption method (ASAP 2460, Micromeritics, USA), and the surface morphology and structure of the materials before and after adsorption were observed by using scanning electron microscope (SEM) and energy dispersive X-ray spectrum (EDS) (ZEISS Gemini 300, OXFORD Xplore, Germany). By Fourier transform infrared spectrometer (FTIR) (Nicolet iS50, Semirfei, USA), 1 mg sample was mixed with 100 mg KBr, pressed into tablets, and scanned in the wavelength range of 450–4000 cm^−1^, which was used to analyze the changes of surface functional groups before and after adsorption. In order to further study the surface morphology and elemental composition of the material, X-ray diffractometer (XRD) (Ultma IV, Rigaku, Japan) was used to analyze the form of the material and determine the crystal structure. The scanning rate was 5 °C/min, and the 2θ range was 10–80°. X-ray photoelectron spectroscopy (XPS) (K-Alpha, Thermo Scientific, USA) was used to study the presence of Cr(VI) on the surface of the material before and after adsorption and the oxidation state of chromium. According to China’s environmental protection standard (GB 7467–87), the concentration of Cr(VI) was determined at 540 nm wavelength based on ultraviolet–visible spectrophotometer (DR1900, Beijing General Instrument, China), and the detection limit was 0.004 mg/L.

### Batch adsorption experiments

All batch tests were carried out in a 100 mL conical flask with a volume of 50 mL, which was sealed by a silicone plug. In addition to study the effect of temperature on this experiment, all other experiments were carried out at room temperature 25 °C using a constant temperature water bath oscillator (SHZ-82B, Jinnan Instruments, China) at 150 rpm. Different parameters were optimized, such as adsorbent dose (1–10 g/L), initial pH (2–10), oscillation time (0–5 h), Cr(VI) concentration (10–110 mg/L) and temperature (25–45 °C). The pH of the solution was adjusted by adding 1 mol/L HCl or NaOH using a pH meter (PHS-3C, Lei, China). At the end of each experiment, the mixture was filtered through a 0.45 μm filter membrane and then the Cr(VI) concentration was quantified. All experiments were conducted in triplicate. The data were expressed as mean ± standard deviation. The test results were recorded and analyzed. The removal rate $R$(%) and adsorption capacity $q_{e}$ (mg/g) of Cr(VI) by WB were calculated according to Eqs. (1)–(2). In addition, detailed information on adsorption kinetics, adsorption isotherms, and adsorption thermodynamic models were provided in the chapters of support material information Sect. 1, Sect. 2 and Sect. 3, respectively.

1 $$ q_{e} = \frac{{\left( {C_{0} - C_{e} } \right)V}}{m} $$

2 $$ R = \frac{{\left( {C_{0} - C_{e} } \right)}}{{C_{0} }} \times 100{\text{\% }} $$

where $C_{0}$ is the initial concentration of Cr(VI), mg/L; $C_{e}$ is the concentration of Cr(VI) in the solution at adsorption equilibrium, mg/L; V is the volume of Cr(VI) solution, L; m is the dosage of biochar, g.

### Experimental model optimization design

As the most commonly used method in RSM, Box–Behnken design (BBD) is the most widely used surface response method because it can accurately describe the linear interaction and quadratic effect of the second-order polynomial model with the least number of experiments, and avoid extreme conditions. In addition, the response surface optimization method also considers the random error of the test. Through the regression fitting process, the response surface and contour are drawn, and the response values corresponding to each factor level can be found. Therefore, we applied it to study and verify the influence of the parameters we selected on the Cr(VI) removal efficiency of WB.

There were five laboratory factors evaluated in this study. Each factor was selected as an independent variable at three levels: initial solution pH (× 1), initial Cr(VI) concentration (× 2), biochar dosage (× 3), adsorption time (× 4), and temperature (× 5). Each independent variable was set at three levels (low, medium, and high, coded as − 1, 0, and + 1, respectively). The level and range of the selected independent variables were given in Table 1. This design illustrated the interaction between independent and dependent variables. The quadratic polynomial equation was used to perform multiple regression on the data from BBD to obtain the parameter estimation of the model (Eq. (3)):

3 $$ y = f\left( x \right) = \beta_{0} + \mathop {\mathop \sum \limits^{k} }\limits_{i = 1} \beta_{i} x_{i} + \mathop {\mathop \sum \limits^{k} }\limits_{i = 1} \mathop {\mathop \sum \limits^{k} }\limits_{j = i + 1} \beta_{ij} x_{i} x_{j} + \mathop {\mathop \sum \limits^{k} }\limits_{i = 1} \beta_{ii} x_{i}^{2} + \varepsilon $$

where $x_{i}$ and $x_{j}$ are coded values; $\beta_{0}$ is constant; $\beta_{i}$ is linear coefficient; $\beta_{ii}$ is quadratic term coefficient; $\beta_{ij}$ is interaction term coefficient; $\varepsilon$ is random error; $k$ is 5 (independent variable value).

**Table 1:** Factors and levels of the Box–Behnken design.

Variables	Symbol	Unit	Variables level		
			Low − 1	Center 0	High + 1
Cr(VI) initial concentration	x_1_	mg/L	30	50	70
BM-WB addition amount	x_2_	g	0.05	0.10	0.15
Reaction time	x_3_	h	1	3	5
Reaction temperature	x_4_	°C	25	35	45
pH	x_5_	–	2	2.5	3

### Reuse test

In order to evaluate the reusability of BM-WB as an adsorbent, the adsorbed BM-WB was added to 50 mL of 0.1 mol/L NaOH solution, and then regenerated by continuous magnetic stirring at 25 °C for 60 min. The regenerated BM-WB was washed three times with ultrapure water to effectively remove the desorbed Cr(VI). The regenerated BM-WB was repeated for 5 adsorption cycles to determine its effectiveness and stability.

## Results and discussion

### Characterization

By comparing the physicochemical properties of WB and BM-WB (SI Table S1), it was found that the ball milling technique could effectively increase the specific surface area and pore volume by 2.87 and 127.77 times, respectively, of WB. In addition, the particle size of biochar could be reduced by ball milling (SI Fig. S1). Based on the analysis of Cr(VI) adsorption by WB and BM-WB (SI Fig. S1), the adsorption amount of Cr(VI) by BM-WB was 3.5–9.1 times higher than that by WB, and BM-WB could provide more binding sites, which could facilitate the exposure of functional groups on the surface of biochar and thus contribute to the effective removal of Cr(VI) from aqueous media^31,32^.

**Figure 1:**
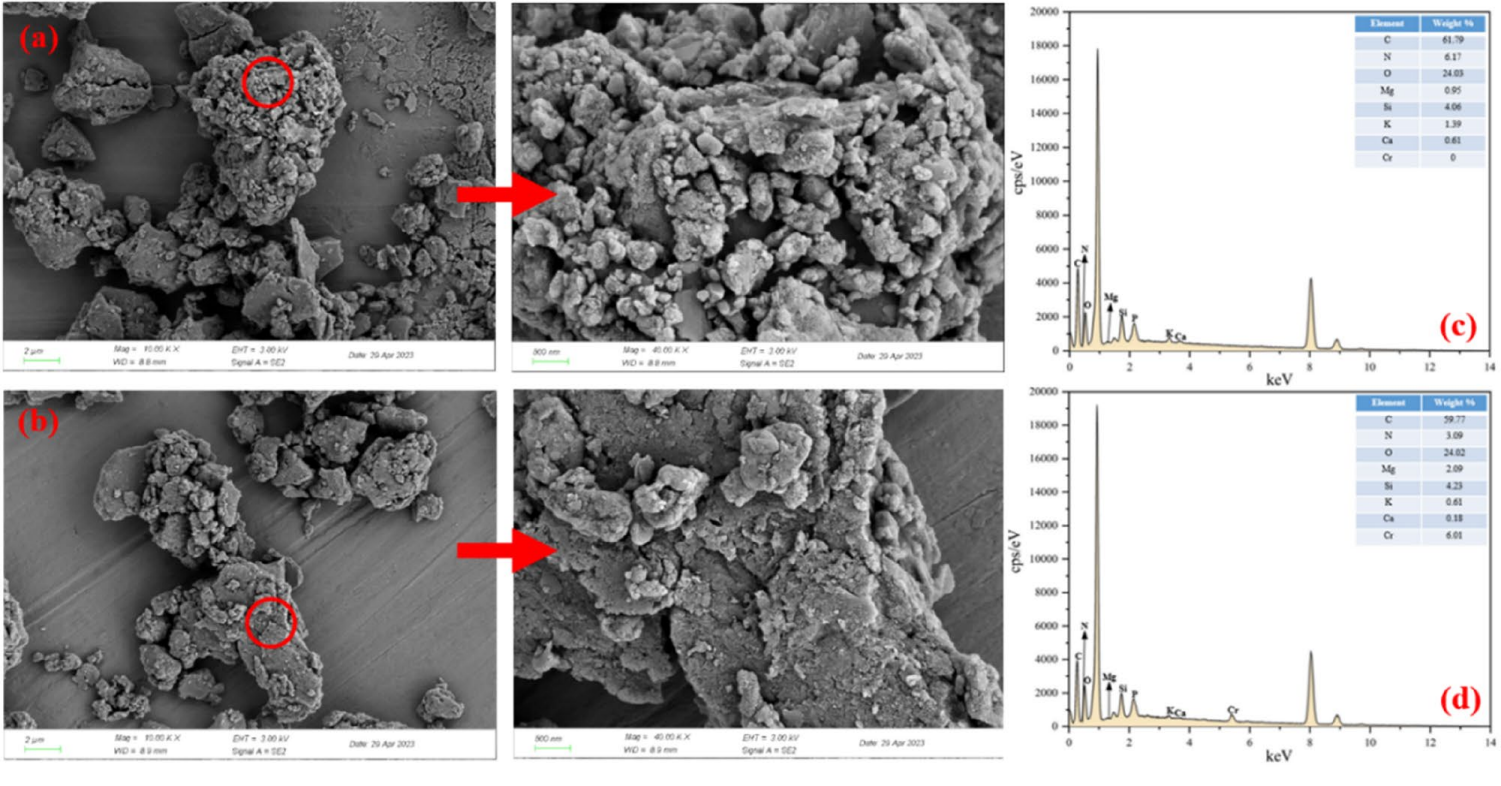
SEM and EDX elemental percentages plots of BM-WB materials: (**a**) before adsorption (10 k × and 40 k ×); (**b**) after adsorption (10 k × and 40 k ×); (**c**) elemental percentages before adsorption; (**d**) elemental percentages after adsorption.

**Figure 2:**
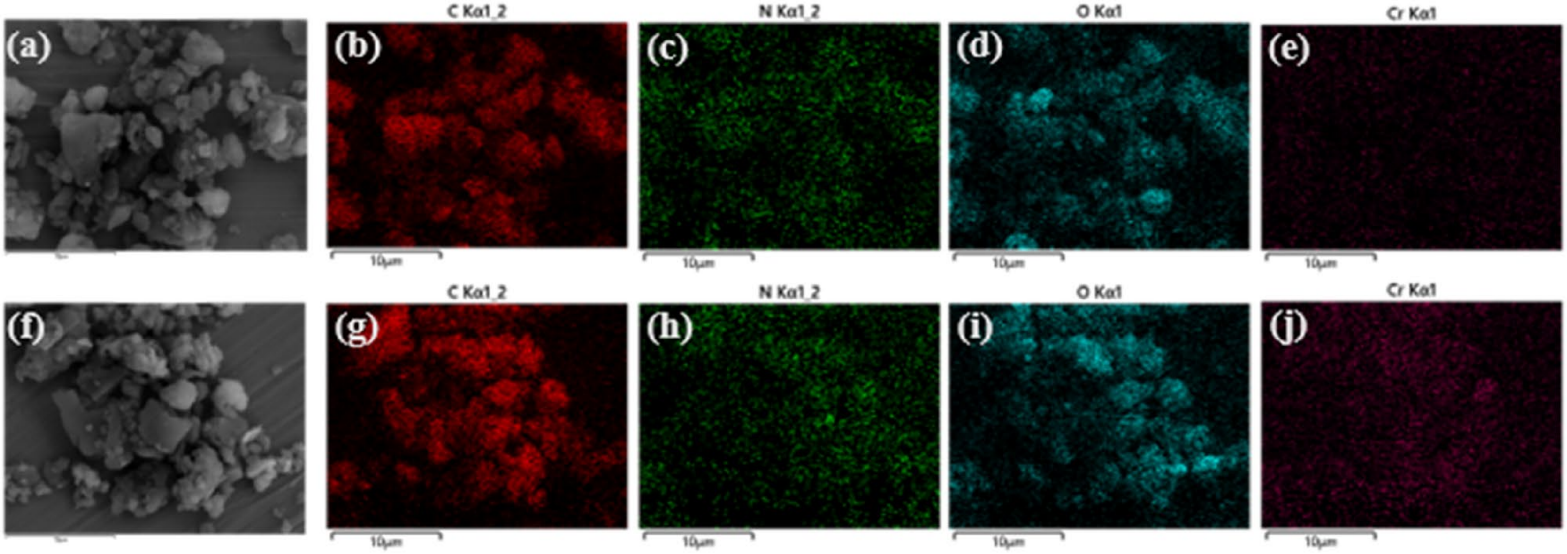
EDX elemental mapping of BM-WB (C, N, O and Cr): before adsorption (**a**–**e**); after adsorption (**f**–**j**).

The morphology and porous structure of BM-WB were characterized by SEM. The structure of BM-WB before adsorption was irregular, with an uneven and rough surface containing macropores and micropores of different shapes, which could provide effective adsorption sites and spaces for the adsorption process (Fig. 1a). The overall surface characteristics of BM-WB tended to be flat after adsorption, and its individual spots showed agglomerated morphology, indicating that the pores were covered by Cr(VI) (Fig. 1b). The elemental composition (Fig. 2 and SI Fig. S2) and content percentages (Fig. 1c and d) of BM-WB were analyzed using EDX, and only after adsorption a Cr elemental content of 6.01% was detected, favorably confirming that Cr(VI) was effectively adsorbed by BM-WB. In addition, significant changes in the elemental contents of Mg, K and Ca occurred before and after adsorption, which could be attributed to the ion-exchange reactions of these metal elements with Cr ions^33^. The changes of C, N and O elements may be attributed to oxidation reactions, complexation reactions and co-precipitation^34^.

**Figure 3:**
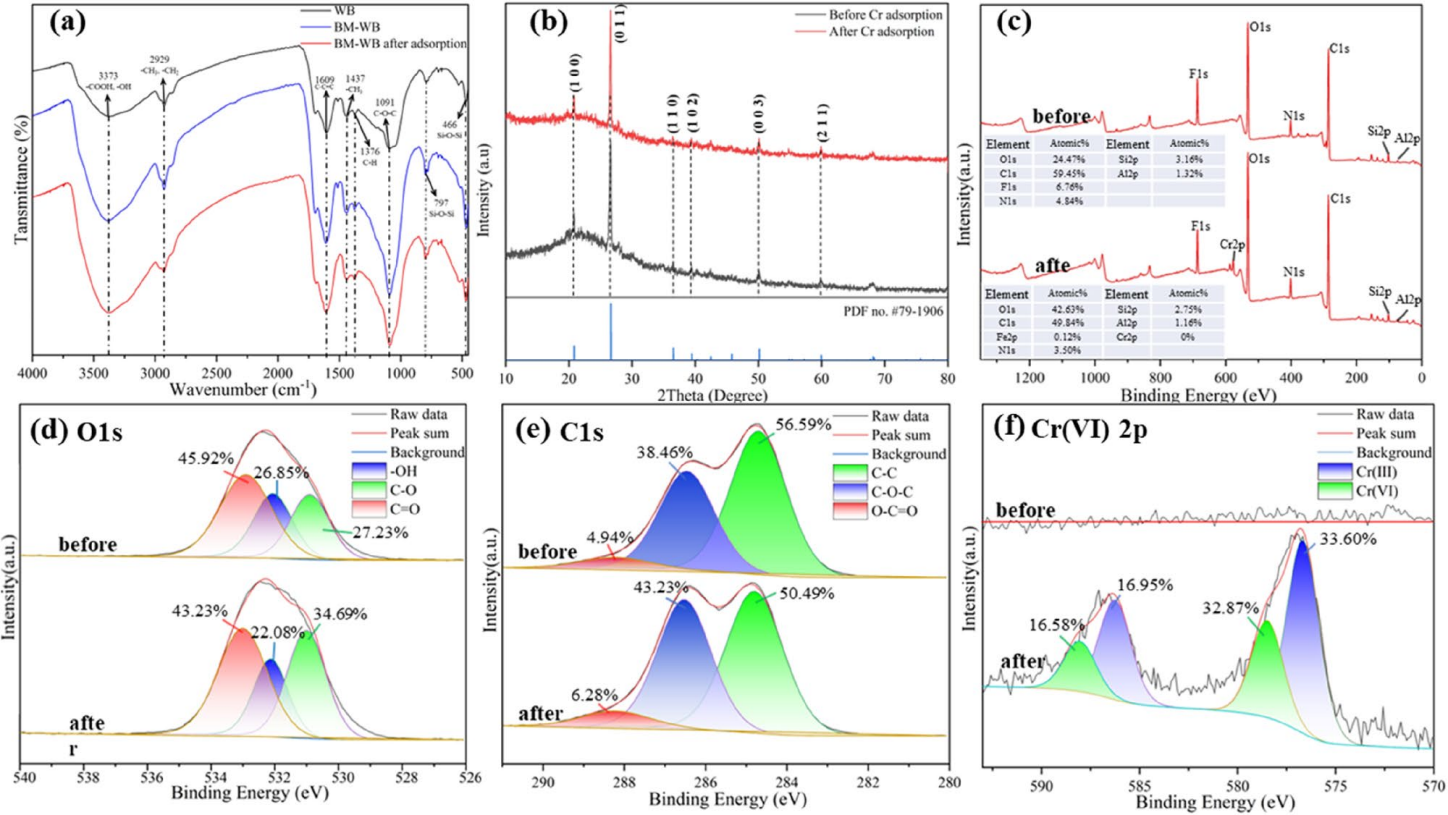
Characterization analysis: (**a**) FTIR; (**b**) XRD; (**c**) XPS full spectrum before and after adsorption; (**d**) Spectrum of O 1 s before and after adsorption; (**e**) Spectrum of C1s before and after adsorption; (**f**) Spectrum of Cr(VI) 2p before and after adsorption.

The main functional groups of the different materials were examined by FTIR (Fig. 3a), and for the WB materials, diffraction peaks were observed at 3373 (–OH), 2929 (–CH_3_, –CH_2_), 1609 (C=C), 1437 (–CH_3_), 1376 (C–H), 1091 (C–O–C), 797 (Si–O–Si) and 466 cm^−1^^35–37^. The diffraction peaks of BM-WB were stretched and the intensity increased significantly, indicating that ball milling can change the content of functional groups^38^. The diffraction peaks of BM-WB were significantly altered before and after adsorption, and the wave number spectral bands and depths became smaller after adsorption, which was attributed to the stretching vibration of the bonds inside the functional groups and the chattering generated during the adsorption of Cr(VI)^39^. Among them, the diffraction peaks at 3373 cm^−1^, 2929 cm^−1^, 1091 cm^−1^ and 466 cm^−1^ were significantly weaker, which indicated that functional groups such as –OH, –CH_3_, C–O–C and Si–O–Si were involved in the Cr(VI) removal reaction.

The crystal structures of BM-WB before and after adsorption were analyzed by XRD (Fig. 3b), and the BM-WB before and after adsorption showed significant peaks at 2θ = 20.8, 26.6, 36.5, 39.5, 50.6, and 60 tilts, corresponding to (100), (011), (110), (102), (003) and (211) crystal planes. The 2θ values before adsorption were 20–40, indicating that only different forms of silica were available. And after adsorption 2θ values = 20.8 and 26.6 had wider peaks, indicating that Cr(VI) was adsorbed on biochar. This is consistent with the phenomenon reported by Kumar et al.^40^.

**Figure 4:**
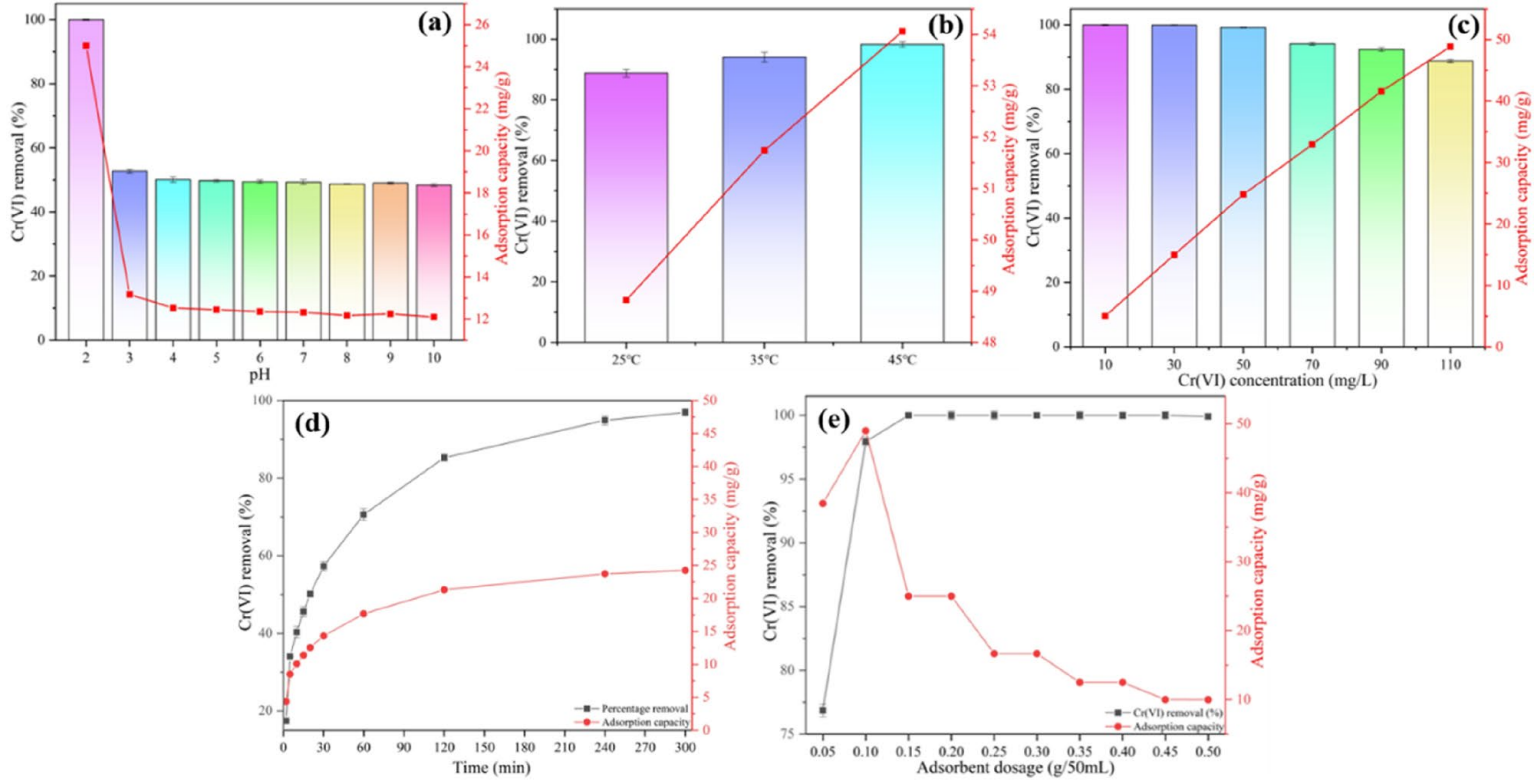
Effects of different parameters: (**a**) solution pH (C_0_ = 50 mg/L, temperature = 25 °C, oscillation speed = 150 rpm, adsorbent dose = 0.1 g, contact time = 5 h); (**b**) Temperature effect (C_0_ = 110 mg/L, oscillation speed = 150 rpm, solution pH = 2.0 ± 0.1, adsorbent dose = 0.1 g); (**c**) Effect of initial concentration (temperature = 25 °C, shaking speed = 150 rpm, solution pH = 2.0 ± 0.1, adsorbent dose = 0.1 g, contact time = 5 h); (**d**) The effect of contact time (temperature = 25 °C, oscillation speed = 150 rpm, solution pH = 2.0 ± 0.1, adsorbent dose = 0.1 g) on the adsorption of Cr(VI) onto BM-WB; (**e**) Effect of adsorbent dose (C_0_ = 50 mg/L, temperature = 25 °C, shaking speed = 150 rpm, solution pH = 2.0 ± 0.1, contact time = 5 h).

The chemical oxidation state and elemental composition of BM-WB were analyzed by XPS method. The changes of the main characteristic peaks on the surface of BM-WB before and after adsorption were O 1 s (+ 2.63%), C 1 s (− 2.41%), F 1s (− 1.1%), N 1s (+ 0.15%), Si 2p (− 0.38%), Al 2p (+ 0.18%), and Cr 2p (+ 0.62%) (Fig. 3c). Before adsorption, no characteristic peak of Cr was detected on the surface of BM-WB, while the presence of the characteristic peak of Cr was clearly detected after adsorption (Fig. 3f), indicating that Cr was successfully adsorbed. The XPS O1s signal of BM-WB was decomposed into three peaks (Fig. 3d), with the binding energy concentrated at 531.0 eV (C–O), 532.1 eV (–OH) and 533.0 eV (C=O). After adsorption, the –OH and O–C=O ratios in BM-WB decreased by 4.77% and 2.69%, respectively, while the C–O increased by 7.23%. In addition, the C1s signal was also decomposed into three peaks (Fig. 3e), with binding energies concentrated at 284.8 eV (C–C), 286.3 eV (C–O–C) and 288.2 eV (C=O). The C–O and –OH characteristic peaks changed significantly after adsorption. It indicated that these functional groups were involved in the adsorption process and their contribution might be related to the electrostatic attraction and complexation reactions occurring in Cr(VI)^41,42^. Further analysis of the different valence states of Cr (Fig. 3f) showed that the Cr 2p peaks at 576.67 and 586.26 eV, 578.48 and 588.05 eV corresponded to Cr(III) and Cr(VI), respectively. The surface molar contents of Cr(III) and Cr(VI) were 50.55% and 49.45%, respectively, confirming that the BM-WB surface adsorption of Cr(VI) might be reduced to Cr(III) by surface functional groups (–COOH, –OH and –NH_2_, etc.)^43^. The XPS analysis results were consistent with the FTIR spectroscopy results (Figs. 3a and 4b).

### BM-WB adsorption study

#### Study of batch tests

**Figure 5:**
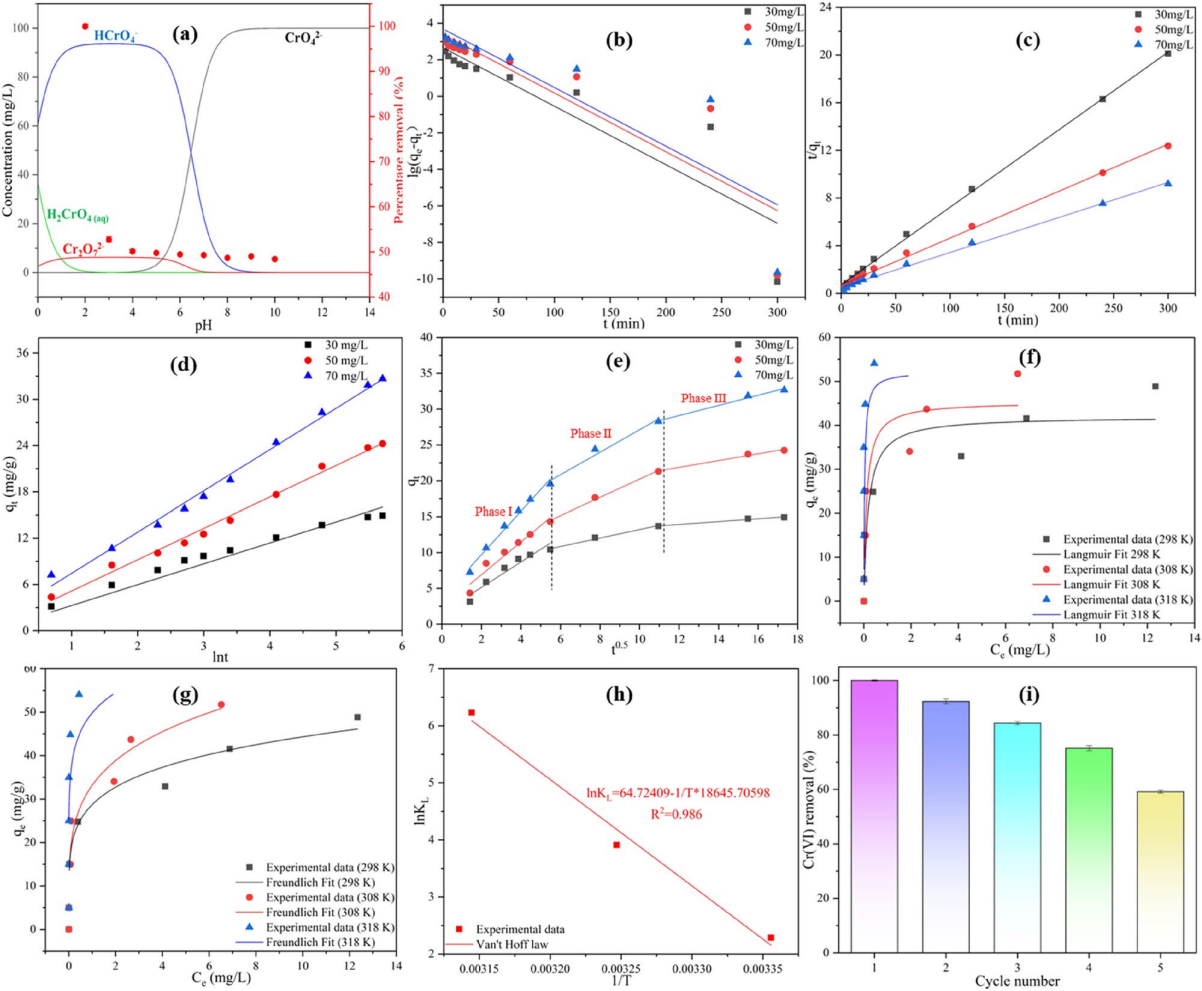
(**a**) Cr(VI) species under different pH conditions and the effect of initial pH on Cr(VI) adsorption; (**b**) Pseudo-first-order rate; (**c**) Pseudo-second-order rate; (**d**) Elovich; (**e**) intraparticle diffusion kinetic model; (**f**) Langmuir; (**g**) Freundlich adsorption isotherm models; (**h**) Van’t Hoff plot for Cr(VI) adsorption onto BM-WB; (**i**) adsorption by BM-WB up to five cycles. (**b**)–(**e**): (temperature = 25 °C, pH = 2.0, BM-WB dosage = 0.1 g); (**f**)–(**g**): (pH = 2.0, BM-WB dosage = 0.1 g, time = 5 h); (**h**): (pH = 2.0, BM-WB dosage = 0.1 g, time = 5 h).

The highest removal rate was achieved at pH 2.0 and decreased sharply at pH > 2 (Fig. 4a), a result that proved that acidic conditions favored the removal of Cr(VI). Cr(VI) in aqueous solution was mainly present in the form of HCrO_4_^−^ (pH = 1.0–5.0) and CrO_4_^2−^ (pH > 8.0) (Fig. 5a). The zero point charge (pH_ZPC_) is strongly influenced by pH^44^. SI Fig. S4 shows the pH of the zero charge point of BM-WB, pH_ZPC_ = 5.62. At pH < pH_ZPC_, the functional groups (–COOH, –OH and –NH_2_, etc.) on the surface of BM-WB are positively charged by protonation, which facilitates the adsorption of HCrO_4_^−^ on the surface of BM-WB by electrostatic attraction^45^. When pH > pH_ZPC_, the functional groups on the surface of BM-WB underwent deprotonation and became negatively charged, and HCrO_4_^−^ might be converted to CrO_4_^2−^ and Cr_2_O_7_^2−^ when using higher pH solutions, due to the negative surface charge and competition with the OH^−^ competition, making BM-WB less attractive to Cr(VI)^46,47^.

The equilibrium adsorption amount of Cr(VI) by BM-WB increased from 48.83 mg/g to 54.06 mg/g when the test temperature was increased from 25 to 45 °C (Fig. 4b). This was because the increase in temperature accelerates the movement of ions in solution, which was favorable for the diffusion of Cr(VI) into the pore channel overcoming the resistance of the BM-WB surface^48,49^. Also, the activation energy to be overcome by the adsorption process was lower at higher temperatures, making the adsorption reaction easier to proceed^50^. Considering the energy-saving perspective, an operating temperature of 25 °C was used for the next experiments.

The equilibrium adsorption of Cr(VI) by BM-WB increased from 5.00 to 48.83 mg/g when the test concentration was increased from 10 mg/L to 110 mg/L (Fig. 4c). The reactive sites on the surface of BM-WB were fully bound to Cr(VI) at high concentrations, while some sites failed to participate in Cr(VI) reduction and adsorption at low concentrations^51^. The adsorption amount of Cr(VI) showed a rapid increase with adsorption time and then stabilized (Fig. 4d), probably because there were more adsorption sites on the BM-WB at the initial stage of the adsorption reaction and the concentration difference between the surface and the solution Cr(VI) was larger, resulting in a larger mass transfer kinetic, and also there was a strong electrostatic attraction between the BM-WB surface and the solution Cr(VI) at this time^52^. The electrostatic attraction between the BM-WB surface and the solution Cr(VI) was also strong at this time^53^. However, as the adsorption proceeds, the adsorption sites were occupied by Cr(VI) and the concentration difference between the surface and solution Cr(VI) decreases gradually, and the electrostatic interaction between the BM-WB surface and Cr(VI) changes from gravitational force to repulsive force, which eventually caused the adsorption rate to stabilize^54^.

The removal of Cr(VI) was positively correlated with the amount of BM-WB dosing, and the removal of Cr(VI) increased from 76.86% to 100% (Fig. 4e). It was shown that the specific surface area of BM-WB exposed to Cr(VI) solution increased with the increase of the dosage, and the number of reaction sites involved in the reduction and adsorption of Cr(VI) increased^55^. To further determine the optimal dosage of the material, the adsorption of Cr(VI) was investigated at different BM-WB dosages (Fig. 4d). The Cr(VI) adsorption amounts were 38.43, 48.97, 25.00, 16.67, 16.67, 12.50, 12.50, 10.00 and 10.00 mg/g at the dosage of 0.05, 0.1, 0.15, 0.2, 0.25, 0.3, 0.35, 0.4, 0.45 and 0.5 g. Among them, the adsorption efficiency was highest when the dosage was 0.1 g, so this optimal dose was used in the next experiments to ensure higher Cr(VI) removal.

#### Adsorption kinetics

The kinetic properties of Cr(VI) adsorption by BM-WB were investigated by fitting first-order, second-order, Elovich and intraparticle models according to the kinetic equations SI Eqs. S (1)–(4), and the model fitting results were shown in Fig. 5b, c, d and e, and the kinetic parameters were shown in SI Tables S2, S3 and S4.

The fitted second order (R^2^ = 0.999) and Elovich model (R^2^ = 0.993) results were better. Among them, the fitted second-order results indicated that the overall rate between BM-WB and Cr(VI) was not only affected by physical diffusion, but the more dominant rate-limiting step was the chemisorption process^56^. Since the large shear force generated during ball milling could improve the surface properties of biochar and increase the number of functional groups, ball milling could enhance the removal of Cr(VI)^57,58^, which was consistent with the phenomenon reported by Zhang et al.^24^. The Elovich model further suggested that the removal of Cr(VI) by BM-WB might be on a homogeneous surface of chemisorption, where the initial concentration had a large effect on the adsorption rate α^59^. The adsorption rate α increased proportionally with increasing initial concentration, which led to an increase in chemisorption rate^60^.

To further elucidate the diffusion mechanism, an intra-particle diffusion model was used (Fig. 5e). q_t_ and t^0.5^ kinetic curves of BM-WB were both straight lines not passing through the origin, indicating that the adsorption process was controlled by multiple steps^61^. The adsorption process could be divided into three stages: liquid film diffusion, intraparticle diffusion and adsorption equilibrium, and k_1_ > k_2_ > k_3_ (SI Table S5), so liquid film diffusion and intraparticle diffusion were the main diffusion rate determining steps^62^.

#### Adsorption isotherm

The adsorption studies of Cr(VI) solutions (10, 30, 50, 70, 90 and 110 mg/L) with different concentrations by BM-WB were carried out using the Langmuir and Freundlich isotherm models (Fig. 5f and g, and the relevant parameters are shown in SI Table S6). The results showed that the data of Cr(VI) adsorption by BM-WB fitted better to the Freundlich model (R^2^ = 1), indicating that the adsorption of Cr(VI) by BM-WB was not monolayer adsorption, but the formation of multilayer adsorbent on the non-uniform surface of BM-WB^63^. This might be attributed to the irregular structure of the BM-WB surface and the properties of O-rich functional groups, which allowed Cr(VI) to form multilayer adsorption on the BM-WB surface and led to a non-uniform distribution of thermal energy^64^.

#### Thermodynamic studies

With the change of solution temperature, the removal rate of Cr(VI) changed obviously. Using the Van’t Hoff law plot (Fig. 5h), $\Delta H^{^\circ }$ and $\Delta S^{^\circ }$ could be determined from the slope and intercept, respectively, and the value of $\Delta G^{^\circ }$ could be further calculated. The calculation of various thermodynamic parameters was shown in Table 2. When the temperature increased from 25 to 45 °C, $\Delta G^{^\circ }$ was negative, and $\Delta G^{^\circ }$ decreased from − 5.67 kJ/mol to − 16.47 kJ/mol. This indicated that the removal of Cr(VI) by BM-WB was spontaneous and the adsorption process was thermodynamically more favorable at higher temperatures^65,66^. The value of $\Delta S^{^\circ }$ was 538.12 kJ/mol/K, indicating that BM-WB had a high affinity for Cr(VI)^40^. The value of $\Delta H^{^\circ }$ was 155.02 kJ/mol, which confirmed that the adsorption process was an endothermic process. With the increase of temperature, q_e_ (mg/g) increased, which was mainly driven by physical and chemical processes such as electrostatic interaction and ion exchange, rather than a single physical or chemical process^67,68^.

**Table 2:** Thermodynamic constants for Cr(VI) adsorption onto BM-WB.

Temp (°C)	Temp (K)	q_e_ (mg/g)	K_L_	$\Delta G^{^\circ }$(KJ/mol)	$\Delta H^{^\circ }$(KJ/mol)	$\Delta S^{^\circ }$(KJ/(mol K))	R^2^
25	298	48.83	9.85	− 5.67	155.02	538.12	0.971
35	308	51.74	50.01	− 10.02			
45	318	54.06	508.33	− 16.47			

The maximum adsorption capacity of BM-WB for Cr(VI) was q_m_ 52.21 mg/g. In comparison with other reported materials for Cr(VI) adsorption, such as Zhou et al. (10.6 mg/g)^69^, Nethaji et al. (57 mg/g)^70^ and so on, the advantages and efficiency of this work were confirmed (SI Table S7). In addition, the adsorption–desorption cycle results of BM-WB are shown in Fig. 5i. The regenerated adsorbent can be reused for at least 5 adsorption–desorption cycles without changing its effectiveness. However, the observed decrease in adsorption efficiency after the third cycle can be attributed to the fact that the surface of BM-WB material was occupied by Cr(VI) during the regeneration process.

### Statistical optimization of BBD and RSM in BM-WB adsorption process

According to the above batch test results, the adsorption conditions were optimized: BM-WB dosage (0.1 g), initial concentration of Cr(VI) (50 mg/L), reaction time (3 h), pH (2.5) and temperature (35 °C) were used as the central points, and BBD was selected for 45 times of optimization experiments. SI Table S8 provided the actual and predicted results of Cr(VI) removal rate. A quadratic polynomial model (Eq. (4)) was established to predict the removal rate of Cr(VI) (*Y*%) :

4 $$ \begin{aligned} Y & = {91}.{48} - {4}.{57}\;{\text{A}} + {5}.{26}\;{\text{B}} + {4}.{87}\;{\text{C}} + {3}.{81}\;{\text{D}} - {19}.{27}\;{\text{E}} + 0.{2525}\;{\text{AB}} + 0.0{35}0\;{\text{AC}} + 0.{6875}\;{\text{AD}} \\ & \quad - 0.{455}0\;{\text{AE}} - 0.{2725}\;{\text{BC}} - 0.{9475}\;{\text{BD}} - {4}.{34}\;{\text{BE}} - 0.{43}00\;{\text{CD}} - 0.{3275}\;{\text{CE}} + 0.{32}00\;{\text{DE}} - 0.{3665}\;{\text{A}}^{{2}} \\ & \quad - {3}.{83}\;{\text{B}}^{{2}} - {2}.{83}\;{\text{C}}^{{2}} - 0.{8298}\;{\text{D}}^{{2}} - {11}.{3}0\;{\text{E}}^{{2}} \\ \end{aligned} $$

The adequacy of the model was determined by different experiments. The naming order model, the sum of squares, and the model summary statistics represented the removal effect of BM-WB on Cr(VI). The results in Tables 3 and 4 showed that the quadratic model fits the experimental data best, with the lowest standard deviation, the highest adjusted R^2^ and predicted R^2^ values, and the lowest P value. Therefore, the quadratic model was used as the best fit for further analysis. Because there were not enough points to estimate the coefficient pairs of the model, the cubic model was not selected.

**Table 3:** Sequential model sum of squares.

Source	Sum of squares	df	Mean square	F-value	p-value	Remarks
Mean versus Total	3.226E + 05	1	3.226E+05			
Linear versus Mean	7330.78	5	1466.16	37.07	< 0.0001	
2FI versus Linear	83.87	10	8.39	0.1667	0.9974	
Quadratic versus 2FI	1193.81	5	238.76	21.63	< 0.0001	Suggested
Cubic versus Quadratic	247.70	15	16.51	8.64	0.0013	Aliased
Residual	17.21	9	1.91			
Total	3.315E+05	45	7365.59

**Table 4:** Model summary statistics.

Source	Std. Dev.	R^2^	Adjusted R^2^	Predicted R^2^	PRESS	Remarks
Linear	6.29	0.8262	0.8039	0.7696	2044.77	
2FI	7.09	0.8356	0.7506	0.5922	3618.53	
Quadratic	3.32	0.9701	0.9453	0.8806	1059.08	Suggested
Cubic	1.38	0.9981	0.9905	0.8775	1087.37	Aliased

**Figure 6:**
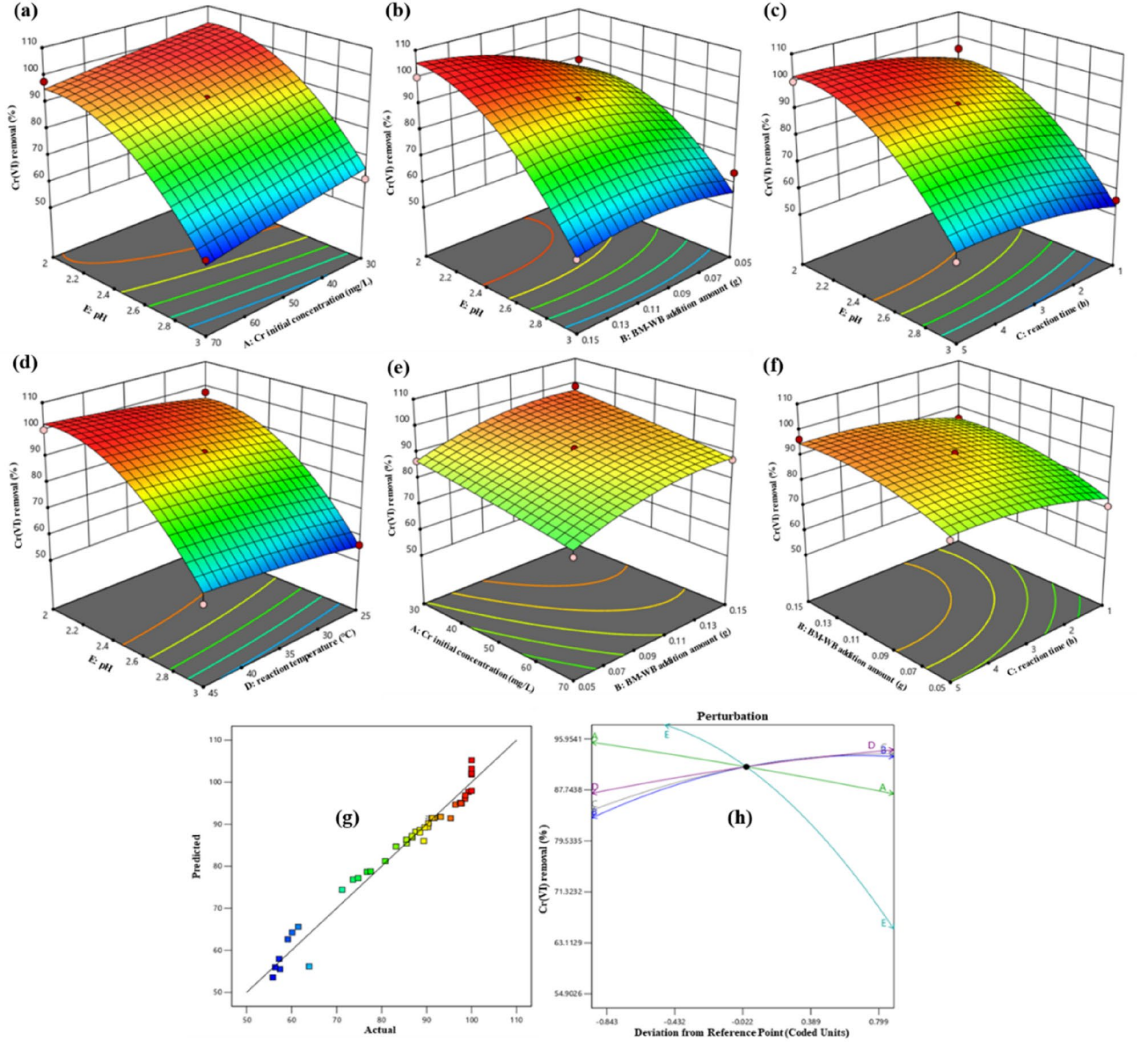
Dual effects of input variables on Cr(VI) removal efficiency (3D plot): (**a**) pH and initial concentration effect; (**b**) pH and dosing amount effect; (**c**) pH and adsorption time effect; (**d**) pH and temperature effect; (**e**) initial concentration and dosing amount effect; (**f**) dosing amount and adsorption time effect; (**g**) predicted and actual values of model on Cr(VI) removal efficiency; (**h**) independent effects of five variables A, B, C, D and E on Cr(VI) removal.

In order to evaluate the reliability of the quadratic model, the F value in the S9 analysis of variance (ANOVA) in the SI table was used for analysis. The F value of the model was 39, and the p value was < 0.001, indicating that the model had a significant effect on the response^71^. Table 5 showed that the R^2^ value of the model was 0.9701, indicating that 97.01% of the model was consistent with the experimental data, and only 2.99% of the data did not match the model. The adjusted R^2^ value was 0.9453, and the R^2^ and the adjusted R^2^ values were close to 1. This confirmed that the model was highly significant under the support of extremely high R^2^ and adjusted R^2^ values^40^. Further analysis of the determination coefficient of the quadratic model in Table 4 showed that the signal-to-noise ratio was measured by ‘sufficient accuracy’. If the value exceeded 4, the model was an ideal model. In this study, the Adeq was 22.7774, which indicated that the signal was sufficient, and this model could be used in the navigation design space^72^. Among them, the CV value was 3.92%, which was far from 10%, further indicating that the precision and reliability of the detection were high. The removal rate obtained by the experiment was compared with the predicted value^68,73^. In Fig. 6g, it could be detected that the value had a high correlation along the uniform distribution of the straight line^29^. It was proved that the model had good applicability to data fitting.

**Table 5:** Determination coefficients in the quadratic model.

Parameter	Value	Parameter	Value
Std. Dev	3.32	R^2^	0.9701
Mean	84.67	Adjusted R^2^	0.9453
C.V. %	3.92	Predicted R^2^	0.8806
		Adeq precision	22.7774

Figure 6 showed the results of various factors and their interactions. When only a single factor was considered (Fig. 6h), the removal rate was positively correlated with BM-WB dosage (B), reaction time (C) and temperature (D), and negatively correlated with initial concentration (A) and pH value, and showed a maximum at the center point. According to the F value in ANOVA, the influence degree of each parameter on adsorption was x_5_ > x_2_ > x_3_ > x_1_ > x_4_, that is, pH had the greatest influence on the removal of Cr(VI). Figure 6a–f were the results of the interaction of various factors. The initial concentration of Cr(VI) (30 mg/L), BM-WB dosage (0.15 g), reaction time (5 h) and temperature (45 °C) were fixed respectively. When the pH was reduced from 3 to 2, the removal rate of Cr(VI) increased by 38.52%, 42.78%, 40.81 and 39.86%, respectively. At a lower pH value, the removal effect of Cr(VI) was better. This was consistent with the above pH batch test results. When the pH value was fixed at 2, the initial concentration of Cr(VI), the dosage of BM-WB, the reaction time and the temperature were changed respectively. It could be found that only by changing the dosage of BM-WB (0.05–0.15 g), the removal efficiency of Cr(VI) was greatly affected (from 89.34% increased to 100%). Maybe with the increase of BM-WB, there were more reaction sites involved in Cr(VI) adsorption, which made it fully combined with Cr(VI) to increase the removal efficiency of Cr(VI).

### Adsorption mechanism

**Figure 7:**
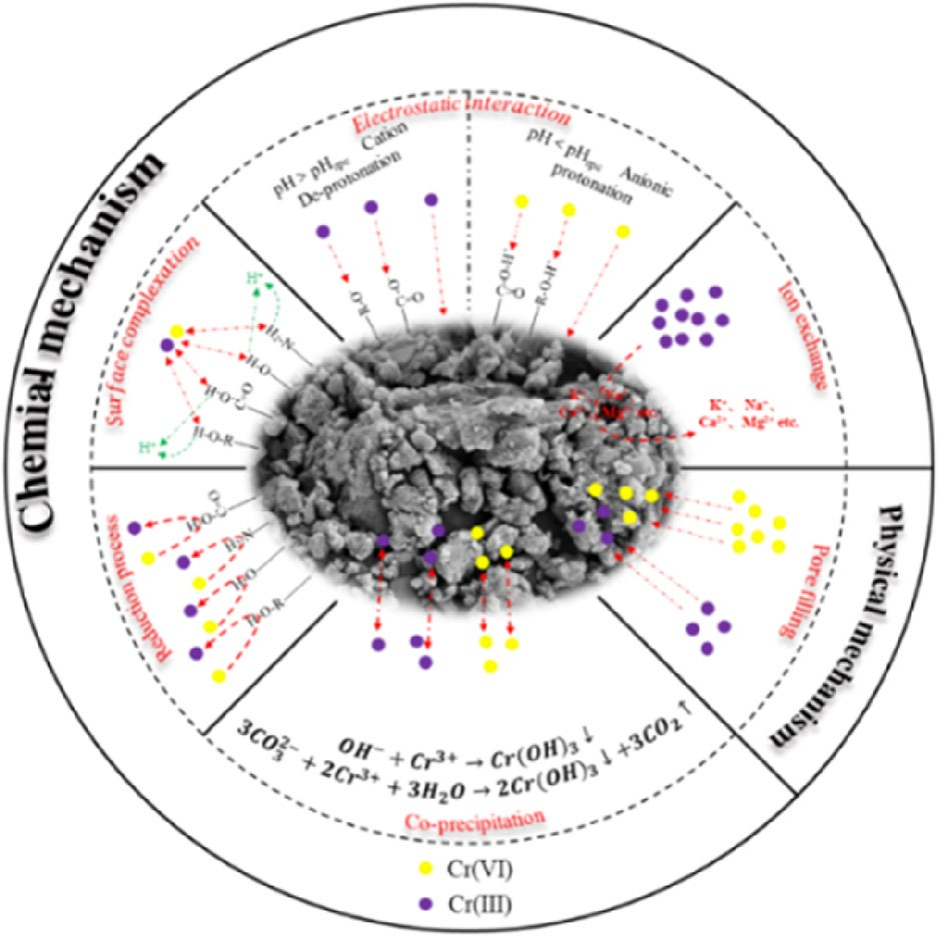
Cr(VI) removal mechanisms by the BM-WB.

The reaction mechanism of Cr(VI) adsorption by BM-WB was analyzed based on the above experimental studies, FTIR and XPS results, and the fitting results of kinetic, isothermal and thermodynamic models (Fig. 7). The adsorption process mainly included the following mechanisms:

(1) Analysis by SEM image showed that the original BM-WB surface structure was irregular and the surface was uneven, containing macropores and micropores of various shapes, providing effective sites and spaces for the adsorption of Cr(VI), and the pores on the surface of BM-WB were covered by Cr(VI) after adsorption filling. Physisorption was the most common and the first reaction to occur, but it was not the main adsorption mechanism for Cr(VI) and was mostly accompanied by other adsorption behaviors at the same time^74^; (2) The pH value of the solution is considered to be an indispensable factor that significantly affects the surface charge state of the adsorbent, the conversion rate of Cr species, and the degree of protonation of functional groups on the adsorbent^75^. Studies have shown that Cr(VI) mainly exists in the form of H_2_CrO_4_, Cr_2_O_7_^2−^, HCrO_4_^−^, and Cr_2_O_4_^2+^ in aqueous solution (Fig. 5a). With the increase of solution pH, the surface charge characteristics of biochar usually change from positive to negative^76^. Many scholars have studied the effect of pH on the adsorption of biochar during the adsorption process. Sun et al.^77^ also determined the contribution of proton exchange by measuring the pH change during the adsorption process, indicating that the decrease of pH value during the adsorption process was accompanied by the release of biochar protons. Xu et al.^78^ also confirmed that it is more obvious at strong acidity (pH = 2) than at weak acidity (pH = 4). When pH > pH_zpc_, the adsorption sites of functional groups (–OH, –COOH and –NH_3_, etc.) on the surface of BM-WB were deprotonated to make the overall surface of biochar negatively charged and electrostatically repelled by HCrO4-ions in the solution, resulting in a decrease in Cr(VI) adsorption and a weak effect on Cr(VI) removal efficiency. When pH < pH_zpc_, the surface functional groups of BM-WB are protonated, and HCrO_4_^−^ ions are attached to the surface of biochar through electrostatic attraction, which is beneficial to the removal of Cr(VI)^24,40^. The response surface experiment also showed that the removal of Cr(VI) by BM-WB was closely related to the pH value; (3) The exchange between cations and heavy metals on the surface of biochar is also one of the important mechanisms of adsorption^79^. XRD analysis showed that the content of original ions (Na^+^, K^+^, Ca^2+^, etc.) on the surface of BM-WB changed significantly before and after adsorption of Cr(VI), which was attributed to the ion exchange of different forms of Cr on BM-WB during adsorption, so that Cr was attached to functional groups rather than original ions^80,81^. The study of Abbas et al.^82^ also showed that C–C, C–O and COO–H of biochar were involved in the exchange of heavy metals. Sun et al.^77^ also confirmed that ion exchange, especially Na+ biochar on the surface of biochar, promoted the removal of heavy metal pollutants by XPS analysis; (4) In addition, the surface of BM-WB is rich in functional groups (–OH, –COOH and –NH_3_, etc.), their H, N, and O, etc., could be used as coordination atoms for coordination complexation with heavy metal ions^83,84^. Qu et al.^85^ also analyzed the changes of C–C, C–O and COO–H after adsorption of Cr(VI) by XPS, indicating that these groups play a leading role in the complex adsorption weight^75^; (5) Since the surface of BM-WB also contained functional groups such as –C–O and –C=O in the form of phenols, ketones and amino groups, these functional groups could be used as electron donors in the process of oxidation to quinone groups and ammonium salts to reduce Cr(VI) to Cr(III)^75^, see Eqs. (5–6). Xu et al.^78^ proved by electron shuttle test that –C–O and –C = O were electron donors for Cr(VI) reduction in biochar when removing Cr(VI) in solution. About 83.5% of Cr adsorbed on biochar was reduced to Cr(III), and the remaining 16.5% was Cr(VI). At the same time, the FTIR spectrum of this study was significantly weaker at the diffraction peak of –OH at 3373 cm^−1^, which further proved that –OH may also be the electron donor part of Cr(VI) reduction; (6) In addition, the surface functional groups can further precipitate with Cr(III) on BM-WB, and can also indirectly affect and promote the coprecipitation behavior through other adsorption mechanisms^86^, see Eqs. (7–8).

5 $$ {\text{Cr}}_{2} {\text{O}}_{7}^{2 - } + 14{\text{H}}^{ + } + 6{\text{e}}^{ - } = 2{\text{Cr}}^{3 + } + 7{\text{H}}_{2} {\text{O}} $$

6 $$ {\text{CrO}}_{4}^{2 - } + 4{\text{H}}_{2} {\text{O}} + 3{\text{e}}^{ - } \to {\text{Cr}}\left( {{\text{OH}}} \right)_{3} + 5{\text{OH}}^{ - } $$

7 $$ {\text{OH}}^{ - } + {\text{Cr}}^{3 + } \to {\text{Cr}}\left( {{\text{OH}}} \right)_{3} \downarrow $$

8 $$ 3{\text{CO}}_{3}^{2 - } + 2{\text{Cr}}^{3 + } + 3{\text{H}}_{2} {\text{O}} \to 2{\text{Cr}}\left( {{\text{OH}}} \right)_{3} \downarrow + 3{\text{CO}}_{2} \uparrow $$

In summary, ball milling can significantly increase the specific surface area of biochar and the content of functional groups, especially the increase of oxygen-containing functional groups plays a leading role in the adsorption of Cr(VI). Among them, electrostatic attraction, surface complexation and redox are the main adsorption mechanisms.

## Conclusions

In this study, BM-WB modified materials with good dispersibility were prepared by ball milling. The effects of BM-WB on the physical and chemical properties of Cr(VI) were compared in detail by various characterization methods. Through the analysis of the adsorption model, the pseudo-second-order model was the best fitting model (R^2^ = 0.999), and the existence of multiple diffusion was confirmed. The maximum adsorption capacity of the Langmuir adsorption isotherm was 52.21 mg/g at 45 °C. The determination of thermodynamic parameters confirmed the endothermic and spontaneous properties of the adsorption process. In addition, the adsorption studies gave various parameters for maximum removal of Cr(VI) under optimized conditions. According to the characterization analysis and experimental results, it is proved that ball milling modification can increase the content of oxygen-containing functional groups, improve the dispersion of surface functional groups, and accelerate the release and transfer of electrons, thus revealing its main removal mechanism. In summary, the preparation process of BM-WB is simple, the cost is low, the adsorption performance is good, and the removal of Cr(VI) in aqueous solution has application potential. Therefore, it has a good application prospect in Cr(VI) pollution remediation. The limitation of this experiment is that it has not been studied in real wastewater. In the follow-up study, we will further explore the interaction and influence mechanism of BM-WB in Cr(VI) co-adsorption system through real wastewater.

### Supplementary Information

Supplementary Information.

## Data Availability

The data used to support the findings of this study are available from the corresponding author upon request.
