# Supplementary material for: Removal of Cr(VI) from aqueous solution using ball mill modified biochar: multivariate modeling, optimization and experimental study
Source: Sci Rep. 2024 Feb 28;14:4853. doi: 10.1038/s41598-024-55520-9 (PMC10901879; doi:10.1038/s41598-024-55520-9)
Supplement: Supplementary file 1 — Supplementary Information. [file 41598_2024_55520_MOESM1_ESM.docx]

Supporting information

**Removal of Cr(VI) from aqueous solution using ball mill modified biochar: multivariate modeling, optimization and experimental study**

Yunfeng Tan ^1, *^, Jinxia Wang ^2, *^, Lingling Zhan ^3^, Hongjun Yang ^4^, Yinchun Gong^5^

^1^ College of River and Ocean Engineering, Chongqing Jiaotong University, Chongqing 400074, China

^2^ College of Resources and Safety, Chongqing Vocational Institute of Engineering, Chongqing 402260, China

^3^ General college, Chongqing Vocational Institute of Engineering, Chongqing 402260, China

^4^ College of Resources and Environment, Southwest University, Chongqing, 400715, China

^5^ Chongqing zhihai technology co., ltd, Chongqing 402260, China

^*^ Corresponding author: **Yunfeng Tan**, e-mail: yunfengtan@126.com; **Jinxia Wang**, e-mail: [jinxiawang@cqvie.edu.cn](mailto:jinxiawang@cqvie.edu.cn)

**Content**

**Section 1.** Adsorption kinetics models.

**Section 2.** Adsorption isotherm models.

**Section 3.** Thermodynamic models.

**Table S1.** Physicochemical properties of WB and BM-WB.

**Table S2.** Pseudo-first-order fitting parameters for simulating Cr(VI) adsorption kinetics data.

**Table S3.** Pseudo-second-order fitting parameters for simulating Cr(VI) adsorption kinetics data.

**Table S4.** Elovich fitting parameters for simulating Cr(VI) adsorption kinetics data.

**Table S5.** Interparticle diffusion fitting parameters for simulating Cr(VI) adsorption kinetics data.

**Table S6.** Comparison of adsorption materials.

**Table S7.** Box-Behnken design of five factors and three levels and the experimental results.

**Table S8.** ANOVA for a quadratic model for the given response of % removal of Cr(VI).

**Fig. S1.** Effect of different materials on Cr(VI) removal ability.

**Fig. S2.** EDX elemental mapping of BM-WB (Mg, Si, K and Ca): before adsorption (a-e); after adsorption (f-j).

**Section 1. Adsorption kinetics** **models.**

Pseudo-first-order (Langmuir,1898), pseudo-second-order (Ho and McKay, 1998b), Elovich (Roginsky and Zeldovich, 1934) and intraparticle diffusion (Weber and Morris, 1963) were used to fit the adsorption data. Pseudo-first-order and pseudo-second-order models are semi-mechanical models based on physical adsorption and chemical adsorption process assumptions, respectively. The Elovich model is an empirical equation for surface heterogeneous chemisorption. The intraparticle diffusion model was used to study the diffusion mechanism of Cr(VI). The model usually includes three steps: liquid film diffusion, intraparticle diffusion and adsorption equilibrium. The equation is as follows:

Pseudo-first-order:

$q_{t}=q_{e}(1-e^{-k_{1}t})$ (1)

Pseudo-second-order:

$q_{t}=\frac{k_{2}q_{e}^{2}t}{1+k_{2}q_{e}t}$ (2)

Elovich model:

$q_{t}=\frac{1}{\beta}ln(\alpha\beta t+1)$ (3)

Intraparticle diffusion:

$q_{t}=k_{i}t^{1/2}+C$ (4)

Where q_e_ and q_t_ (mg/g) are the adsorption capacities at equilibrium and time t (min), respectively; *k*_1_ (1/min) and *k*_2_ (g/(mg·min) are the adsorption rate constants of the pseudo-first-order and pseudo-second-order kinetic models, respectively; *α* (mg/(g·min)) and *β* (g/mg) are the initial adsorption rate and the desorption constant, respectively; ki (mg/(g·min^0.5^)) is the rate constant of the intraparticle diffusion model, *C* (mg/g) is a constant related to the thickness of the boundary layer, and the higher value of *C* corresponds to the greater effect of the limiting boundary layer.

**Section 2. Adsorption isotherm models.**

In order to understand and analyze the data, Langmuir (Langmuir,1918) and Freundlich (Freundlich,1906) models were used to fit the adsorption data. The Langmuir model is used to describe the monolayer adsorption on the homogeneous surface, and the Freundlich model is suitable for the chemical adsorption on the heterogeneous surface. The equation is as follows:

Langmuir model:

$q_{e}=\frac{{q_{m}K}_{L}C_{e}}{1+K_{L}C_{e}}$ (5)

Freundlich model:

$q_{e}=K_{F}C_{e}^{1/n}$ (6)

where *q_e_* is the equilibrium Cr(VI) uptake (mg/g), *C_e_* is the aqueous Cr(VI) concentration (mg/L), *q_m_* is the maximum adsorption capacity of BM-WB (mg/g), *K_L_* is the Langmuir constant (L/mg), *K_F_* is the Freundlich affinity coefficient (mg/g(L/mg)^1/n^), *n* is the exponential coefficient.

**Section 3. Thermodynamic models.**

The values of important thermodynamic parameters such as entropy change (Δ*S°* (J/(mol·K))), Gibbs free energy change (Δ*G°* (kJ/mol)) and enthalpy change (Δ*H°* (kJ/mol)) were determined to understand the nature of the adsorption process. The equation is as follows (Egbosiuba et al. 2020):

$K_{d}=\frac{q_{e}}{C_{e}}$ (7)

${lnK}_{d}=\frac{{\Delta S}^{0}}{R}-\frac{{\Delta H}^{0}}{RT}$ (8)

${\Delta G}^{0}=-RTlnK_{d}={\Delta H}^{0}-T{\Delta S}^{0}$ (9)

where *K_d_* is the partition coefficient, defined as the ratio of the equilibrium adsorption volume q_e_ (mg/g) to the equilibrium concentration *C_e_* (mg/L); *ρ* (g/L) is the liquid density; *R* is the gas constant (*R*＝8.314 J/(mol·K)); and *T* (K) is the adsorption temperature in Kelvin.

**Table S1.** Physicochemical properties of WB and BM-WB

| **Sample** | **WB** | **BM-WB** |
| --- | --- | --- |
| BET specific surface area (m^2^/g) | 38.39 | 110.08 |
| Pore size (nm) | 3.4474 | 0.116341 |
| Pore volume (cm^3^/g) | 0.033087 | 4.2274 |

**Table S2.** Pseudo-first-order fitting parameters for simulating Cr(VI) adsorption kinetics data

| **initial concentration (mg/L)** | **Pseudo-first-order dynamic model** | | | **standard error of intercept** | **standard error of slope** |
| --- | --- | --- | --- | --- | --- |
|  | ***q_e_ (mg/g)*** | ***K*_1_ (min^-1^）** | ***R*^2^** |  |  |
| 30 | 14.34 | 0.032 | 0.814 | 0.70046 | 0.00542 |
| 50 | 29.99 | 0.032 | 0.785 | 0.77146 | 0.00597 |
| 70 | 40.05 | 0.032 | 0.768 | 0.80591 | 0.00623 |

**Table S3.** Pseudo-second-order fitting parameters for simulating Cr(VI) adsorption kinetics data

| **initial concentration (mg/L)** | **Pseudo-second-order dynamic model** | | | **standard error of intercept** | **standard error of slope** |
| --- | --- | --- | --- | --- | --- |
|  | ***q_e_ (mg/g)*** | ***K*_2_ (mg/g·min）** | ***R*^2^** |  |  |
| 30 | 15.38 | 0.006 | 0.999 | 0.08176 | 0.00063 |
| 50 | 25.38 | 0.002 | 0.997 | 0.09472 | 0.00073 |
| 70 | 34.03 | 0.002 | 0.997 | 0.07087 | 0.00055 |

**Table S4.** Elovich fitting parameters for simulating Cr(VI) adsorption kinetics data

| **initial concentration (mg/L)** | **Elovich dynamic model** | | | **standard error of intercept** | **standard error of slope** |
| --- | --- | --- | --- | --- | --- |
|  | ***α*** | ***β*** | ***R*^2^** |  |  |
| 30 | 3.325 | 0.369 | 0.944 | 0.86577 | 0.23263 |
| 50 | 5.302 | 0.246 | 0.992 | 0.47771 | 0.12836 |
| 70 | 7.917 | 0.187 | 0.993 | 0.58865 | 0.15817 |

**Table S5.** Interparticle diffusion fitting parameters for simulating Cr(VI) adsorption kinetics data

| **initial concentration (mg/L)** | **k_1_ (mg/(g·min^0.5^))** | **C** | **R^2^** | **k_2_ (mg/(g·min^0.5^))** | **C** | **R^2^** | **k_3_ (mg/(g·min^0.5^))** | **C** | **R^2^** |
| --- | --- | --- | --- | --- | --- | --- | --- | --- | --- |
| 30 | 1.771 | 1.603 | 0.928 | 0.506 | 7.313 | 0.988 | 0.198 | 11.555 | 0.981 |
| 50 | 2.285 | 2.343 | 0.948 | 1.267 | 7.548 | 0.995 | 0.473 | 16.187 | 0.986 |
| 70 | 3.026 | 3.652 | 0.986 | 1.569 | 11.426 | 0.974 | 0.709 | 20.583 | 0.989 |

**Table S6.** Adsorption isotherm parameters for Cr(VI) adsorption by BM-WB

| **Temp (℃)** | **Langmuir** | | |  | **Freundlich** | | |
| --- | --- | --- | --- | --- | --- | --- | --- |
|  | **q_m_ (****mg/g)** | **K_L_ (L/mg)** | **R^2^** |  | **K_F_ (mg/g(L/mg)^1/n^)** | **n** | **R^2^** |
| 25 | 41.99 | 5.155 | 0.655 |  | 28.734 | 5.314 | 0.957 |
| 35 | 45.35 | 8.295 | 0.826 |  | 33.178 | 4.393 | 0.924 |
| 45 | 52.21 | 28.473 | 0.883 |  | 49.803 | 7.685 | 1 |

**Table S7.** Comparison of adsorption materials

| **Adsorbent** | **Initial Cr(VI) concentration (mg/L)** | **pH** | **Dosage (g/L)** | **Reaction time (h)** | ***q_m_* (mg/g)** | **Reference** |
| --- | --- | --- | --- | --- | --- | --- |
| Fe@Fe_2_O_3_  Core-Shell Nanowires | 8 | 6.5 | 1.0 | 5 | 7.8 | (Ai et al. 2008) |
| Biochar-supported zero valent iron | 53 | 5.7 | 2 | 24 | 10.6 | (Zhou et al. 2014) |
| Iron-containing bamboo charcoal | 50 | 5.0 | 2.0 | 24 | 33 | (Wang et al. 2011) |
| Magnetic Fe_3_O_4_-FeB | 8 | 6.3 | 0.8 | 5 | 38.9 | (Shen et al. 2016) |
| Magnetite-coated actived carbon | 50-125 | 2.0 | 1.0 | 5 | 57 | (Nethaji et al. 2013) |
| BM-WB | 110 | 2.0 | 2.0 | 5 | 52.21 | This work |

**Table S8.** Box-Behnken design of five factors and three levels and the experimental results

| **Run** | **Variables and their coded values** | | | | | | | | | | **Cr removal (%)** | | $\boldsymbol{\varepsilon}$ |
| --- | --- | --- | --- | --- | --- | --- | --- | --- | --- | --- | --- | --- | --- |
|  | **Cr(Ⅵ) initial concentration (mg/L)** | | **BM-WB addition amount (g)** | | **Reaction time (h)** | | **Reaction temperature (℃)** | | **pH** | | **Y_exp_** | **Y_pre_** |  |
|  | **x_1_** | **Code x_1_** | **x_2_** | **Code x_2_** | **x_3_** | **Code x_3_** | **x_4_** | **Code x_4_** | **x_5_** | **Code x_5_** |  |  |  |
| 1 | 50 | 0 | 0.15 | 1 | 3 | 0 | 35 | 0 | 3 | +1 | 57.22 | 58.00 | 0.78 |
| 2 | 50 | 0 | 0.1 | 0 | 3 | 0 | 45 | +1 | 3 | +1 | 60.14 | 64.21 | 4.07 |
| 3 | 50 | 0 | 0.05 | -1 | 3 | 0 | 35 | 0 | 2 | -1 | 89.34 | 86.02 | 3.32 |
| 4 | 50 | 0 | 0.15 | +1 | 1 | -1 | 35 | 0 | 2.5 | 0 | 85.55 | 85.48 | 0.07 |
| 5 | 50 | 0 | 0.1 | 0 | 5 | +1 | 45 | +1 | 2.5 | 0 | 98.55 | 96.06 | 2.49 |
| 6 | 30 | -1 | 0.1 | 0 | 3 | 0 | 35 | 0 | 2 | -1 | 100.00 | 103.2 | 3.2 |
| 7 | 50 | 0 | 0.1 | 0 | 5 | +1 | 25 | -1 | 2.5 | 0 | 90.30 | 89.30 | 1 |
| 8 | 50 | 0 | 0.05 | -1 | 5 | +1 | 35 | 0 | 2.5 | 0 | 83.20 | 84.69 | 1.49 |
| 9 | 50 | 0 | 0.1 | 0 | 5 | +1 | 35 | 0 | 3 | +1 | 59.18 | 62.62 | 3.44 |
| 10 | 50 | 0 | 0.1 | 0 | 3 | 0 | 35 | 0 | 2.5 | 0 | 91.74 | 91.48 | 0.26 |
| 11 | 50 | 0 | 0.1 | 0 | 3 | 0 | 35 | 0 | 2.5 | 0 | 91.31 | 91.48 | 0.17 |
| 12 | 30 | -1 | 0.1 | 0 | 1 | -1 | 35 | 0 | 2.5 | 0 | 88.53 | 88.01 | 0.52 |
| 13 | 30 | -1 | 0.1 | 0 | 3 | 0 | 25 | -1 | 2.5 | 0 | 93.13 | 91.73 | 1.4 |
| 14 | 70 | 1 | 0.1 | 0 | 5 | +1 | 35 | 0 | 2.5 | 0 | 88.48 | 88.61 | 0.13 |
| 15 | 50 | 0 | 0.1 | 0 | 1 | -1 | 35 | 0 | 3 | +1 | 55.83 | 53.53 | 2.3 |
| 16 | 50 | 0 | 0.1 | 0 | 1 | -1 | 45 | +1 | 2.5 | 0 | 86.75 | 87.18 | 0.43 |
| 17 | 30 | -1 | 0.1 | 0 | 3 | 0 | 45 | +1 | 2.5 | 0 | 100.00 | 97.97 | 2.03 |
| 18 | 70 | +1 | 0.15 | +1 | 3 | 0 | 35 | 0 | 2.5 | 0 | 87.59 | 88.23 | 0.64 |
| 19 | 50 | 0 | 0.1 | 0 | 3 | 0 | 35 | 0 | 2.5 | 0 | 91.16 | 91.48 | 0.32 |
| 20 | 70 | +1 | 0.1 | 0 | 3 | 0 | 25 | -1 | 2.5 | 0 | 80.78 | 81.22 | 0.44 |
| 21 | 50 | 0 | 0.1 | 0 | 3 | 0 | 35 | 0 | 2.5 | 0 | 91.59 | 91.48 | 0.11 |
| 22 | 70 | +1 | 0.1 | 0 | 3 | 0 | 45 | +1 | 2.5 | 0 | 90.40 | 90.21 | 0.19 |
| 23 | 50 | 0 | 0.05 | -1 | 1 | -1 | 35 | 0 | 2.5 | 0 | 71.22 | 74.41 | 3.19 |
| 24 | 30 | -1 | 0.1 | 0 | 3 | 0 | 35 | 0 | 3 | +1 | 61.48 | 65.57 | 4.09 |
| 25 | 70 | +1 | 0.05 | -1 | 3 | 0 | 35 | 0 | 2.5 | 0 | 74.78 | 77.20 | 2.42 |
| 26 | 50 | 0 | 0.1 | 0 | 3 | 0 | 25 | -1 | 3 | +1 | 56.35 | 55.95 | 0.4 |
| 27 | 50 | 0 | 0.15 | +1 | 5 | +1 | 35 | 0 | 2.5 | 0 | 96.44 | 94.67 | 1.77 |
| 28 | 50 | 0 | 0.15 | +1 | 3 | 0 | 25 | -1 | 2.5 | 0 | 89.58 | 89.22 | 0.36 |
| 29 | 30 | -1 | 0.1 | 0 | 5 | +1 | 35 | 0 | 2.5 | 0 | 99.31 | 97.68 | 1.63 |
| 30 | 30 | -1 | 0.05 | -1 | 3 | 0 | 35 | 0 | 2.5 | 0 | 86.80 | 86.84 | 0.04 |
| 31 | 50 | 0 | 0.05 | -1 | 3 | 0 | 35 | 0 | 3 | +1 | 63.93 | 56.16 | 7.77 |
| 32 | 50 | 0 | 0.15 | +1 | 3 | 0 | 35 | 0 | 2 | -1 | 100.00 | 105.23 | 5.23 |
| 33 | 50 | 0 | 0.15 | +1 | 3 | 0 | 45 | +1 | 2.5 | 0 | 97.64 | 94.94 | 2.7 |
| 34 | 50 | 0 | 0.1 | 0 | 3 | 0 | 25 | -1 | 2 | -1 | 97.49 | 95.14 | 2.35 |
| 35 | 70 | +1 | 0.1 | 0 | 3 | 0 | 35 | 0 | 3 | +1 | 57.42 | 55.52 | 1.9 |
| 36 | 70 | +1 | 0.1 | 0 | 1 | -1 | 35 | 0 | 2.5 | 0 | 77.56 | 78.81 | 1.25 |
| 37 | 50 | 0 | 0.1 | 0 | 1 | -1 | 25 | -1 | 2.5 | 0 | 76.78 | 78.71 | 1.93 |
| 38 | 50 | 0 | 0.05 | -1 | 3 | 0 | 25 | -1 | 2.5 | 0 | 73.66 | 76.80 | 3.14 |
| 39 | 50 | 0 | 0.05 | -1 | 3 | 0 | 45 | +1 | 2.5 | 0 | 85.51 | 86.31 | 0.8 |
| 40 | 50 | 0 | 0.1 | 0 | 1 | -1 | 35 | 0 | 2 | -1 | 95.33 | 91.42 | 3.91 |
| 41 | 50 | 0 | 0.1 | 0 | 3 | 0 | 35 | 0 | 2.5 | 0 | 91.59 | 91.48 | 0.11 |
| 42 | 50 | 0 | 0.1 | 0 | 5 | +1 | 35 | 0 | 2 | -1 | 99.99 | 101.82 | 1.83 |
| 43 | 30 | -1 | 0.15 | +1 | 3 | 0 | 35 | 0 | 2.5 | 0 | 98.60 | 96.86 | 1.74 |
| 44 | 50 | 0 | 0.1 | 0 | 3 | 0 | 45 | +1 | 2 | -1 | 100.03 | 102.11 | 2.08 |
| 45 | 70 | +1 | 0.1 | 0 | 3 | 0 | 35 | 0 | 2 | -1 | 97.76 | 94.98 | 2.78 |

Yexp and Ypre are experimental and predicted responses.

**Table S9.** ANOVA for a quadratic model for the given response of % removal of Cr(VI).

| **Source** | **Sum of squares** | $\boldsymbol{df}$ | **Mean square** | ***F* value** | **P value**  **Prob＞*F*** |  |
| --- | --- | --- | --- | --- | --- | --- |
| Model | 8608.47 | 20 | 430.42 | 39.00 | < 0.0001 | significant |
| X_1__ (mg·L^-1^) | 333.79 | 1 | 333.79 | 30.24 | < 0.0001 |  |
| X_2__ (g·L^-1^) | 442.89 | 1 | 442.89 | 40.13 | < 0.0001 |  |
| X_3__ (h) | 379.28 | 1 | 379.28 | 34.36 | < 0.0001 |  |
| X_4__ ℃ | 231.95 | 1 | 231.95 | 21.01 | 0.0001 |  |
| X_5__ pH | 5942.87 | 1 | 5942.87 | 538.41 | < 0.0001 |  |
| X_1_X_2_ | 0.2550 | 1 | 0.2550 | 0.0231 | 0.8805 |  |
| X_1_X_3_ | 0.0049 | 1 | 0.0049 | 0.0004 | 0.9834 |  |
| X_1_X_4_ | 1.89 | 1 | 1.89 | 0.1713 | 0.6826 |  |
| X_1_X_5_ | 0.8281 | 1 | 0.8281 | 0.0750 | 0.7865 |  |
| X_2_X_3_ | 0.2970 | 1 | 0.2970 | 0.0269 | 0.8711 |  |
| X_2_X_4_ | 3.59 | 1 | 3.59 | 0.3253 | 0.5737 |  |
| X_2_X_5_ | 75.43 | 1 | 75.43 | 6.83 | 0.0152 |  |
| X_3_X_4_ | 0.7396 | 1 | 0.7396 | 0.0670 | 0.7980 |  |
| X_3_X_5_ | 0.4290 | 1 | 0.4290 | 0.0389 | 0.8454 |  |
| X_4_X_5_ | 0.4096 | 1 | 0.4096 | 0.0371 | 0.8489 |  |
| X_1_^2^ | 1.09 | 1 | 1.09 | 0.0990 | 0.7557 |  |
| X_2_^2^ | 119.33 | 1 | 119.33 | 10.81 | 0.0031 |  |
| X_3_^2^ | 65.38 | 1 | 65.38 | 5.92 | 0.0228 |  |
| X_4_^2^ | 5.60 | 1 | 5.60 | 0.5076 | 0.4831 |  |
| X_5_^2^ | 1038.19 | 1 | 1038.19 | 94.06 | < 0.0001 |  |
| Residual | 264.91 | 24 | 11.04 |  |  |  |
| Lack of fit | 264.68 | 20 | 13.23 | 237.30 | < 0.0001 | not significant |
| Pure error | 0.2231 | 4 | 0.0558 |  |  |  |
| Cor total | 8873.38 | 44 |  |  |  |  |


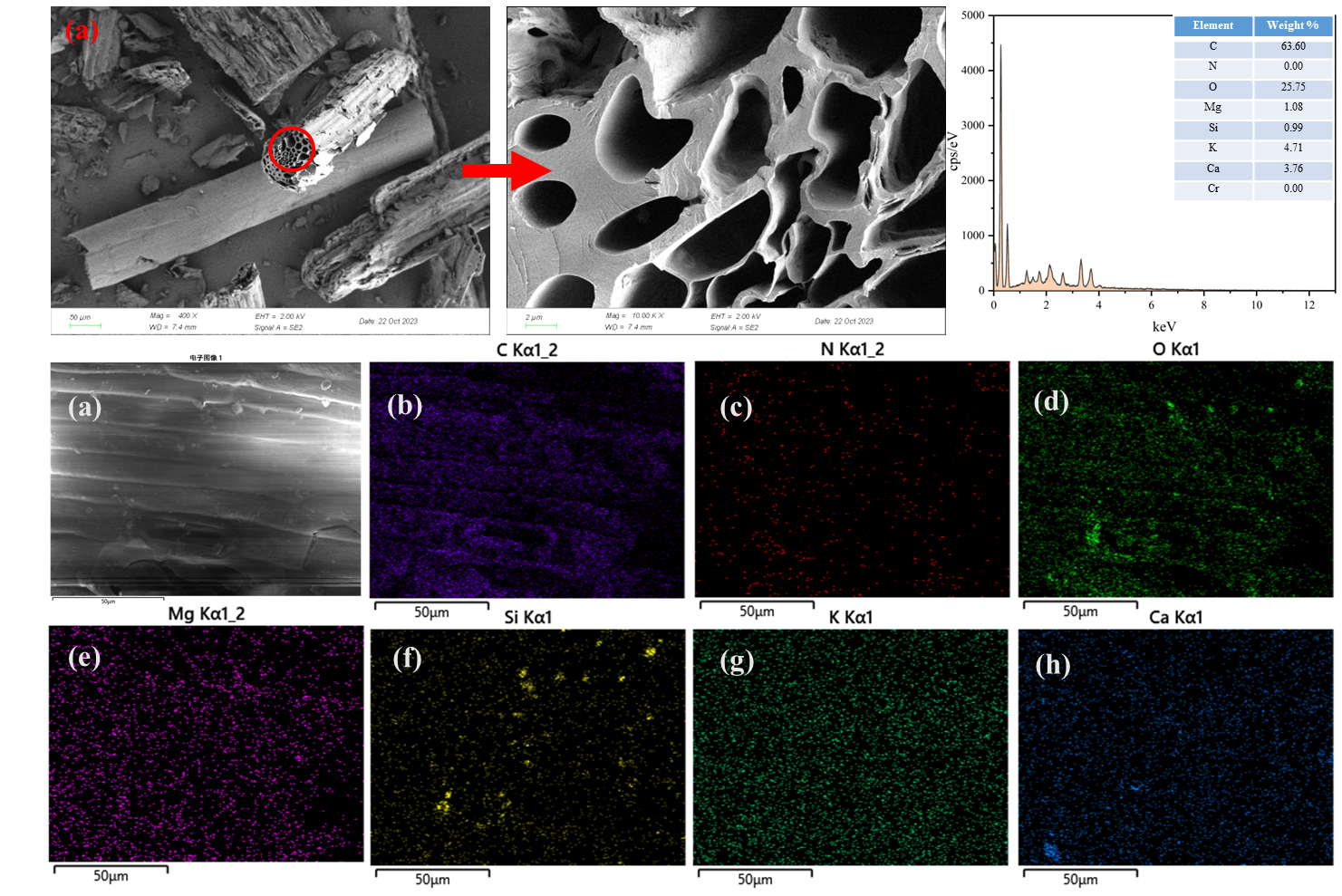


**Fig. S1.** SEM-EDX of WB


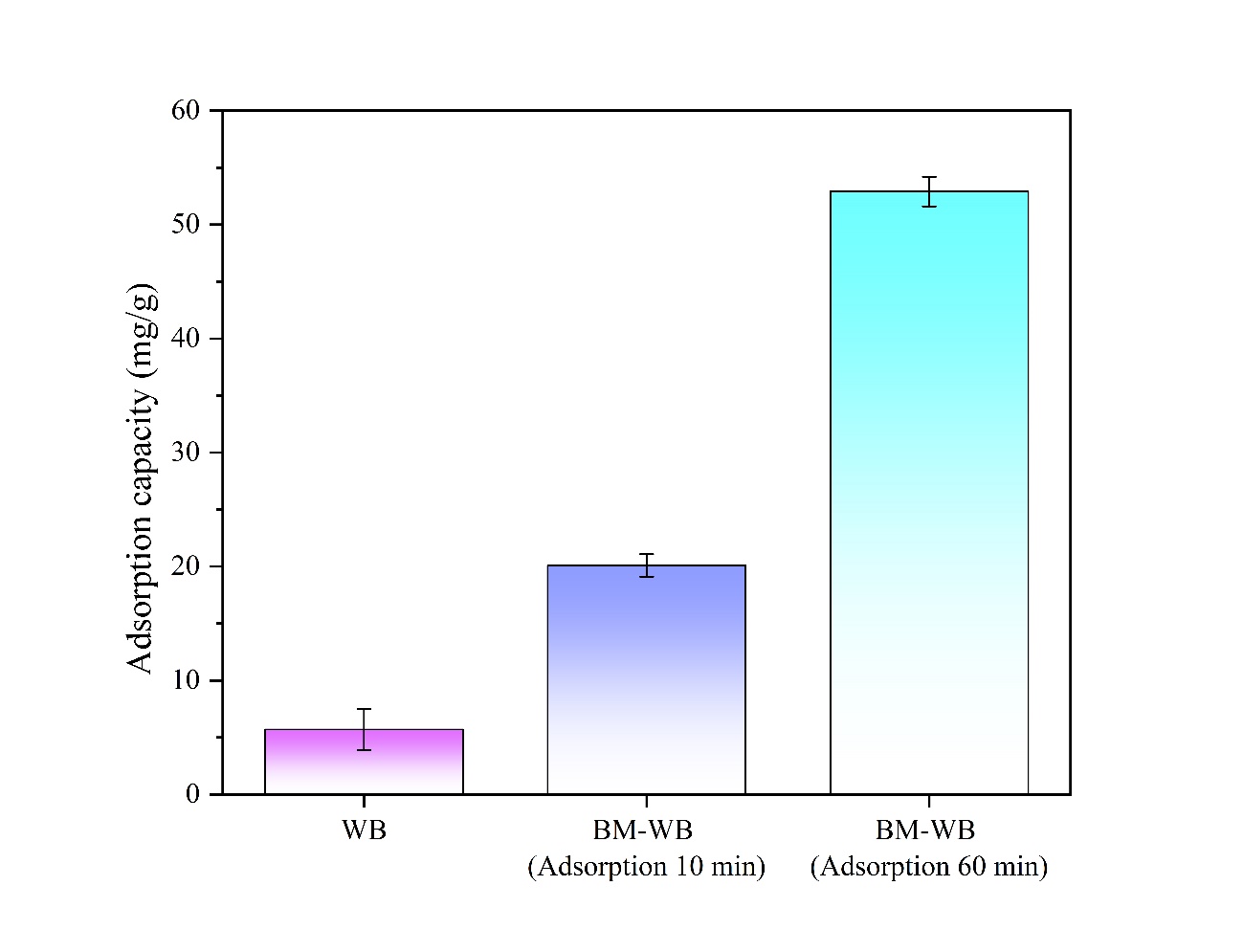


**Fig. S2.** Effect of different materials on Cr ( VI ) removal ability.


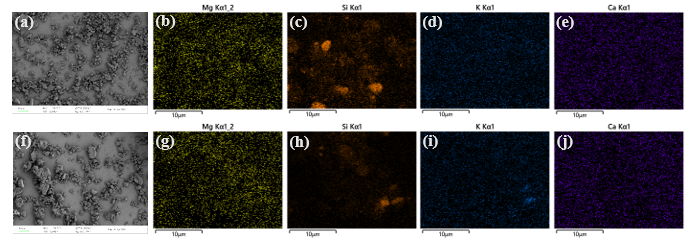


**Fig. S3.** EDX elemental mapping of BM-WB (Mg, Si, K and Ca): before adsorption (a-e); after adsorption (f-j).

**
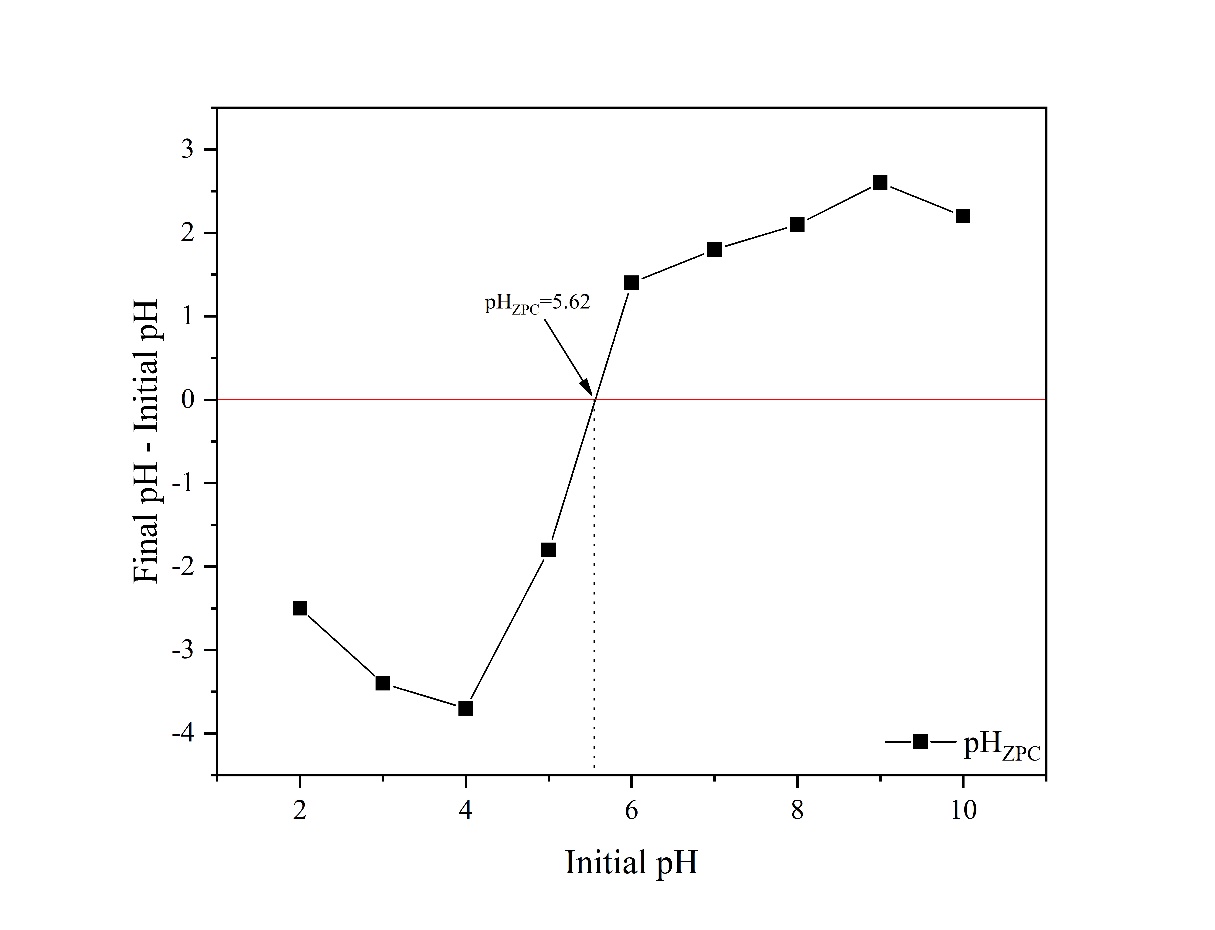
**

**Fig. S4.** pH_ZPC_ of the BM-WB

**References**

Ai Z, Cheng Y, Zhang L, Qiu J (2008) Efficient removal of Cr(VI) from aqueous solution with Fe@Fe2O3 core-shell nanowires. Environmental Science & Technology 42 (18): 6955-6960

Egbosiuba TC, Abdulkareem AS, Kovo AS, Afolabi EA, Tijani JO, Auta M, Roos WD (2020) Ultrasonic enhanced adsorption of methylene blue onto the optimized surface area of activated carbon: Adsorption isotherm, kinetics and thermodynamics. Chemical Engineering Research & Design 153: 315-336

Freundlich, H., 1906. Over the adsorption in solution. J. Phys. Chem 57, 1100-1107.

Ho, Y.S., McKay, G., 1998b. Sorption of dye from aqueous solution by peat. Chemical Engineering Journal 70, 115-124.

Lagergren, S.K., 1898. About the theory of so-called adsorption of soluble substances. Sven. Vetenskapsakad. Handingarl 24, 1-39.

Langmuir, I., 1918. The adsorption of gases on plane surfaces of glass, mica and platinum. Journal of the American Chemical Society 40, 1361-1403.

Nethaji S, Sivasamy A, Mandal AB (2013) Preparation and characterization of corn cob activated carbon coated with nano-sized magnetite particles for the removal of Cr(VI). Bioresource Technology 134: 94-100

Roginsky, S., Zeldovich, Y.B., 1934. The catalytic oxidation of carbon monoxide on manganese dioxide. Acta Phys. Chem. USSR 1, 2019.

Shen W, Mu Y, Xiao T, Ai Z (2016) Magnetic Fe3O4-FeS nanocomposites with promoted Cr(VI) removal performance. Chemical Engineering Journal 285: 57-68

Wang XJ, Wang Y, Wang X, Liu M, Xia SQ, Yin DQ, Zhang YL, Zhao JF (2011) Microwave-assisted preparation of bamboo charcoal-based iron-containing adsorbents for Cr(VI) removal. Chemical Engineering Journal 174 (1): 326-332

Weber, W.J., Morris, J.C., 1963. Kinetics of adsorption on carbon from solution. Journal of the sanitary engineering division 89, 31-60.

Zhou Y, Gao B, Zimmerman AR, Chen H, Zhang M, Cao X (2014) Biochar-supported zerovalent iron for removal of various contaminants from aqueous solutions. Bioresource Technology 152: 538-542
